# Cognitive flexibility deficits differ between the sexes in a transchromosomic model of Down syndrome

**DOI:** 10.1242/dmm.053030

**Published:** 2026-10-28

**Authors:** Elysabeth Otte, Nicole Gordon, Andrew Folz, Charles R. Goodlett, Randall J. Roper

**Affiliations:** ^1^ Department of Biology, Indiana University Indianapolis, Indianapolis, IN 46202, USA; ^2^ Department of Psychology, Indiana University Indianapolis, Indianapolis, IN 46202, USA; ^3^ Stark Neurosciences Research Institute, Indiana University School of Medicine, Indianapolis, IN 46202, USA

**Keywords:** Trisomy 21, Transchromosomic, Cognitive flexibility, Development, Sex differences, Behavior

## Abstract

Individuals with Down syndrome (DS) exhibit cognitive and behavioral phenotypes, including intellectual disability and reduced cognitive flexibility. The TcMAC21 mouse model of DS [Tc(HSA21,CAG-EGFP)1Yakaz/J] has a freely segregating human chromosome 21 containing ∼93% of protein-coding genes and was hypothesized to show significant deficits in behavioral, learning and memory tests. Examination of male and female TcMAC21 and control mice from postnatal day (P)45 to P62 showed that TcMAC21 mice had lower body weight than controls, with greater differences in males than in females. Locomotor activity testing on P45 revealed that TcMAC21 mice had greater activity than controls early in the session and increased time in the arena center later in the session, and a longer stride length in gait analysis. In the Morris water maze, TcMAC21 mice did not differ significantly from euploid littermates in acquisition of either cued or place training, although male (but not female) TcMAC21 mice showed significant deficits during reversal, providing the first demonstration of sex-specific impairment of cognitive flexibility in a mouse model of DS. These findings support the hypothesis that, in young adults with DS, males exhibit more severe deficits in cognitive flexibility than females.

## Teaser

**Editor's choice:** The transchromosomic TcMAC21 mouse model of Down syndrome exhibits changes in locomotor activity and gait, and sex-specific alterations in cognitive flexibility, compared to euploid controls.

## INTRODUCTION

Down syndrome (DS) is the most common live-born aneuploidy and the leading genetic cause of intellectual disability (Antonarakis et al., 2020). DS is caused by the triplication of human chromosome 21 (Hsa21), leading to a variety of phenotypes, including developmental delays, enduring challenges in domains of cognitive, motor, communication and executive function, skeletal abnormalities and heart defects (Antonarakis et al., 2020; Thomas and Roper, 2021). These phenotypes profoundly impact daily living and pose significant challenges for individuals with DS (Lott and Dierssen, 2010; Russo et al., 2024; Schworer et al., 2023). To better understand the genetic underpinnings of these phenotypes and explore mechanisms and potential therapeutic interventions, preclinical studies involving experimental animal models are needed that achieve construct, face and predictive validity when translated to phenotypes in individuals with DS.

Transchromosomic rodent models containing DNA from a human chromosome may provide a novel avenue for understanding the phenotypes associated with developmental disorders such as DS. A new transchomosomic model, the Tc(HSA21,CAG-EGFP)1Yakaz/J (TcMAC21) mouse, was created with a freely segregating Hsa21q-mouse artificial chromosome (MAC) (Kazuki et al., 2020). This hybrid chromosome contains ∼93% of the genes located on Hsa21q (∼26 Mb Hsa21q sequences), attached to a mouse chromosome 11 centromere. The inclusion of a copy of the human Hsa21 genes to produce triploidy in this model was done with the hopes of improving construct validity and enabling modeling of most DS phenotypes. The TcMAC21 mouse model of DS is not mosaic; does not contain the duplications, rearrangements and sequence alterations seen in the Tc1 transchromosomic model (O'Doherty et al., 2005; Gribble et al., 2013); and has been reported to model many DS phenotypes, including cardiac anomalies, craniofacial alterations, and molecular and cellular pathologies (Kazuki et al., 2020). In addition, the TcMAC21 model does not contain triplicated non-orthologous genes to Hsa21, as seen in the Ts65Dn model, and encompasses more of the protein-coding genes from Hsa21 than most other models of DS, including Dp(16)1Yey, which includes ∼65% of the mouse orthologs to Hsa21. In TcMAC21 mice, 106 of 117 mouse orthologs found on Hsa21 were expressed at levels that were 80-120% of levels measured in euploid littermates. Protein-coding genes from Hsa21 showed transcription levels that were, on average, about half the transcript levels that were quantified from mouse orthologs. Knowledge is limited about the extent to which the Hsa21q genes are properly translated into proteins that function in murine systems. Previous studies have identified deficits in cerebellar circuit functioning, delayed neurogenesis, and impairments in learning, memory and synaptic plasticity (Kazuki et al., 2020; Stander et al., 2026; Kurabayashi et al., 2023). Inclusion of TcMAC21 mice in studies of DS may help to improve preclinical translation, offering a humanized platform for testing drug efficacy and human-specific gene therapies.

The present study of the TcMAC21 mouse model of DS expands previous work by examining cognitive phenotypes during late adolescence and early adulthood in both sexes. The purpose of this study was to build upon the behavioral profile of previous data collected from this novel TcMAC21 mouse model using a series of tests of cognitive and behavioral phenotypes with translational significance for DS. The tasks (spatial learning and reversal; locomotor activity in a novel arena; balance and gait) are sensitive to (and deficits are associated with) structural or functional impairments in neural circuits in the cerebellum, hippocampal formation or frontal cortex, brain regions vulnerable to neurodevelopmental disruption in DS (Lott and Dierssen, 2010; Russo et al., 2024). For some instances in which the main cognitive phenotypes inform behaviors associated with individuals with DS, including cognitive deficits and learning disabilities, we highlight key sex differences that may be influencing the development of the phenotypes in these individuals. Assessment of these phenotypes in young-adult TcMAC21 mice of both sexes provided an important contribution to the characterization of this novel mouse model and the extent to which it may model the human DS condition, with potential for advancing preclinical therapeutic interventions to individuals with DS.

## RESULTS

### Body weights

Body weight measurements were taken at the beginning of the testing period (P45), at the start of water maze testing (P51) and again at the conclusion of testing (P62) (Fig. 1). Weights significantly increased with age [main effect of age, F(2,116)=133.78, *P*<0.001; η^2^_p_=0.70], and males weighed more than females [main effect of sex, F(1,58)=31.61, *P*<0.001; η^2^_p_=0.353]. Euploid mice weighed more than TcMAC21 mice [main effect of genotype, F(1,58)=47.04, *P*<0.001; η^2^_p_=0.448], and the magnitude of that difference increased significantly with age [genotype×age interaction, F(2,116)=15.17, *P*<0.001; η^2^_p_=0.207]. The weight differences between euploid and TcMAC21 mice were significantly larger in males than in females [genotype×sex interaction, F(1,58)=6.504, *P*=0.013; η^2^_p_=0.101]. Follow-up repeated measures ANOVAs (RM-ANOVAs) were performed for each sex separately. For males, TcMAC21 mice weighed significantly less than euploid littermates [main effect of genotype, F(1,36)=43.62, *P*<0.001; η^2^_p_=0.548], and those group differences increased significantly with age [genotype×age interaction, F(2,72)=10.28, *P*<0.001; η^2^_p_=0.222]. Simple main effects comparisons indicated that euploid male mice weighed significantly more than TcMAC21 mice at each age examined (*P*<0.001); the euploid group showed significant weight increases from P45 to P51 and from P51 to P62 (*P*<0.001, pairwise comparisons with Bonferroni corrections), whereas the TcMAC21 group only showed significant weight increases from P45 to P51 (*P*<0.001), not from P51 to P62 (*P*=1.00). Similarly for females, TcMAC21 mice weighed significantly less than euploid counterparts [main effect of genotype, F(1,22)=17.67, *P*<0.001; η^2^_p_=0.445], and those group differences increased significantly with age [genotype×age interaction, F(2,44)=7.532, *P*=0.0014; η^2^_p_=0.258]. Simple main effects comparisons indicated that euploid female mice weighed significantly more than TcMAC21 female mice at each age examined (P45, *P*=0.013; P51, *P*=0.003; P62, *P*<0.001); the euploid group showed significant weight increases from P45 to P51 (*P*<0.001) and P51 to P62 (*P*<0.001), whereas the TcMAC21 group only showed significant weight increases from P45 to P51 (*P*<0.001), not from P51 to P62 (*P*=0.49). Thus, TcMAC21 mice of both sexes showed reduced rates of growth from P45 to P62 compared to those of euploid littermates (average percentage increases, respectively, of 16.2% versus 9.4% in males and of 16.7% versus 9.5% in females).

**Fig. 1.**
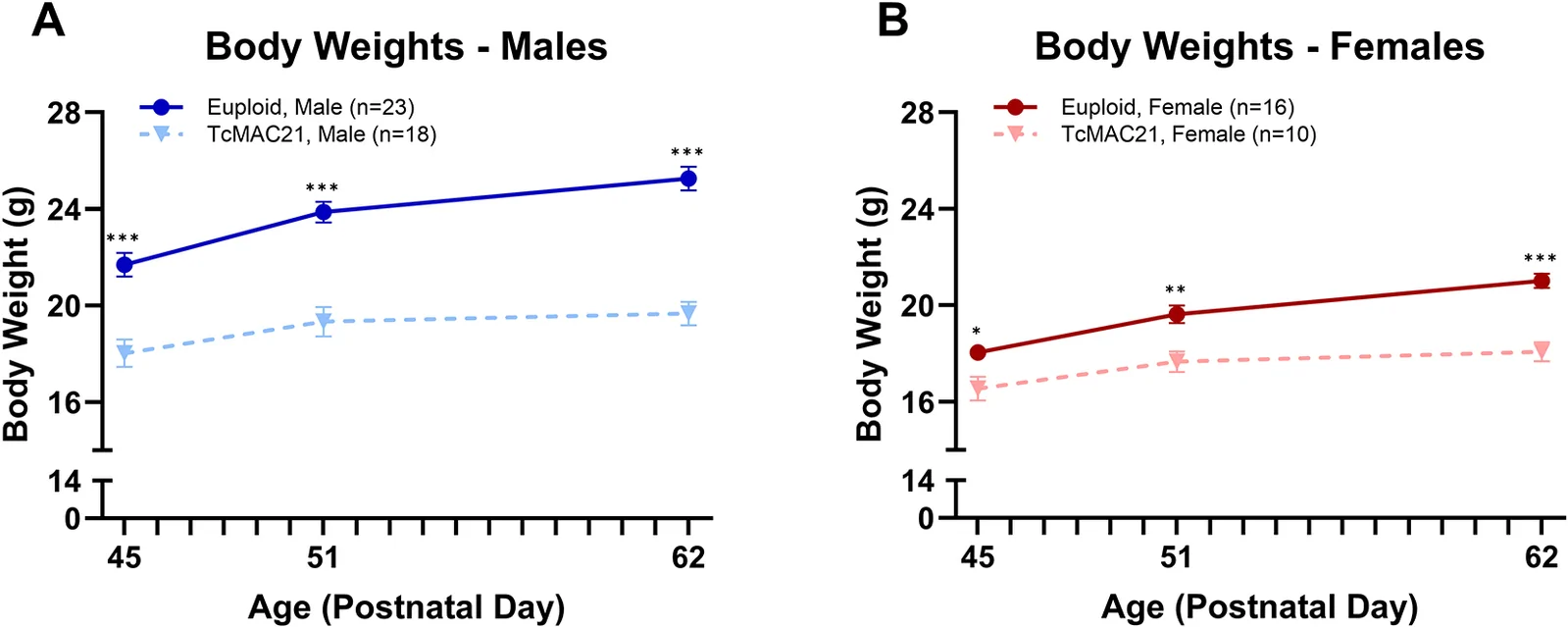
**Body weights of euploid and TcMAC21 male and female mice.** (A,B) During the testing window [postnatal day (P)45-P62], male (A) and female (B) TcMAC21 animals weighed significantly less than same-sex euploid littermates at each age, and the differences between euploid and TcMAC21 groups of the same sex increased significantly with age in both sexes. *P<0.05; **P<0.01; ***P<0.001; simple effects comparisons of genotype at each age within sex. Euploid, male (*n*=23); TcMAC21, female (*n*=18); euploid, female (*n*=16); TcMAC21, female (*n*=10).

These reduced rates of growth from adolescence to young adulthood in TcMAC21 mice are consistent with previous findings showing that birthweights of TcMAC21 pups do not differ from those of euploid littermates, but weight deficits and growth restriction emerge by the second postnatal week, persist to P35 and are even magnified by 3 months of age (Sarver et al., 2023; Stander et al., 2026; Kazuki et al., 2020). These growth deficits are also observed in other mouse models of DS, including Ts65Dn, in which significantly smaller body sizes have been reported in trisomic animals, compared to those of euploid controls, often contributing to early postnatal death (Roper et al., 2006).

### Locomotor activity

All groups showed the expected reductions in distance traveled over the session [main effect of time bin, F(14.749,781.678)=22.734, *P*<0.001; η^2^_p_=0.300; Fig. 2A,C]. Activity patterns of TcMAC21 and euploid mice differed over the session [significant time bin×genotype interaction, F(14.749,781.678)=1.711, *P*=0.045; η^2^_p_=0.031; there were no main or interactive effects of sex]. Simple effects contrasts between genotypes at each time bin (sexes combined) indicated that TcMAC21 mice were more active than euploid littermates early in the session (Fig. 2E, sexes combined), reaching significance at 2 and 4 min (*P*<0.05), but activity levels and rates of decline were comparable thereafter.

**Fig. 2.**
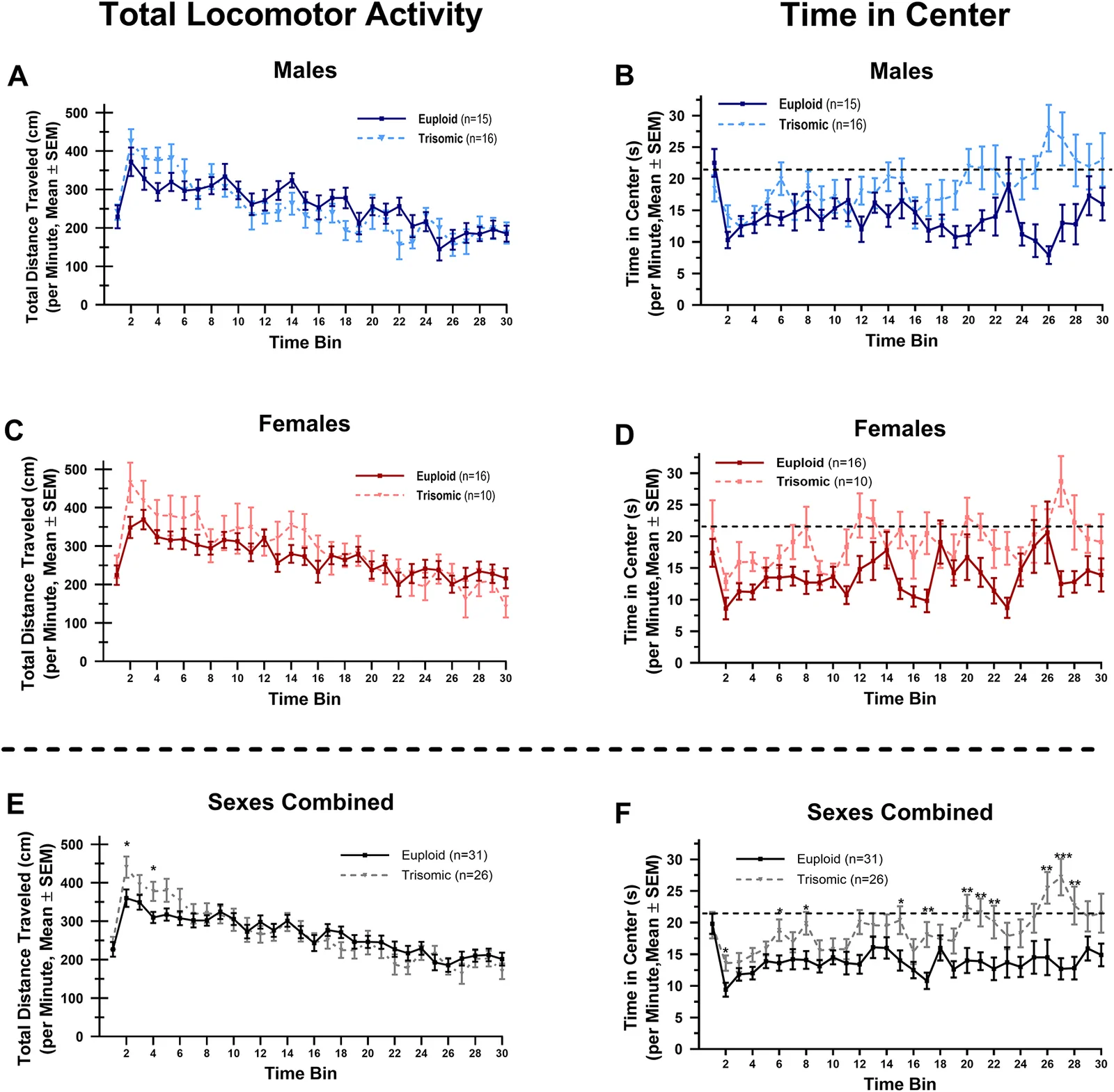
**Locomotor activity of TcMAC21 male and female mice and euploid littermates.** (A-D) Locomotor activity shown as a function of total distance traveled per 1-min time bins (A,C) and time spent in center per 1-min time bins (B,D) over a 30-min testing period in males (A,B) and females (C,D). Dashed lines represent the expected value of time in the center for each time bin if activity was distributed randomly between center and periphery, based on the proportional surface area of the two. (E,F) The same data as in A-D, with sexes combined for follow-up analyses to highlight the significant genotype×time bin interaction in both measures (total locomotor activity, E; time in center, F). Total activity decreased over time, and the TcMAC21 mice were more active than euploid mice early in the session (genotype×time bin interaction, *P*=0.045). For time in center, there were striking differences between genotypes over the session, in which TcMAC21 mice spent significantly more time in the center than euploid littermates, and those differences increased significantly over time (genotype×time bin interaction, *P*=0.033). *P<0.05; **P<0.01; ***P<0.001; simple main effects comparisons between genotypes (sexes combined) for each time bin. Euploid, male (*n*=15); TcMAC21, male (*n*=16); euploid, female (*n*=16); TcMAC21, female (*n*=10).

In contrast, differences between TcMAC21 and euploid mice in time spent in the center were evident across the session in both sexes and became more prominent in the second half (Fig. 2B,D). The TcMAC21 animals spent significantly more time in the center than did euploid controls [main effect of genotype, F(1,53)=14.882, *P*<0.001; η^2^_p_=0.219], and the differences between genotypes grew larger over time [time bin×genotype interaction, F(13,181,698.599)=1.830, *P*=0.034; η^2^_p_=0.033], albeit in different patterns in males and females over time [time bin×genotype×sex interaction, F(13,181,698.599)=1.769, *P*=0.043; η^2^_p_=0.032], as shown in Fig. 2B,D. As shown in Fig. 2F (sexes combined to highlight the time bin×genotype interaction), simple effects contrasts between genotypes at each time bin indicated that TcMAC21 mice spent significantly more time in the center than did euploid littermates in 4 of the first 15 min and in 7 of the last 15 min of the session.

To facilitate comparisons of the time-in-center data of our locomotor activity test to data reported in the original TcMAC21 study in their open-field test (Kazuki et al., 2020), we converted the data from Fig. 2B,D to the same scales reported by Kazuki and colleagues in their fig. 6d (Kazuki et al., 2020; i.e. to percentage time-in-center per 5-min time bins) and plotted in Fig. 3. The data in Fig. 3 tend to have overall higher percentage time scores than those of Kazuki and colleagues, but the genotype differences in males and females are nevertheless comparable (compare Fig. 3 with fig. 6d of Kazuki et al., 2020).

**Fig. 3.**
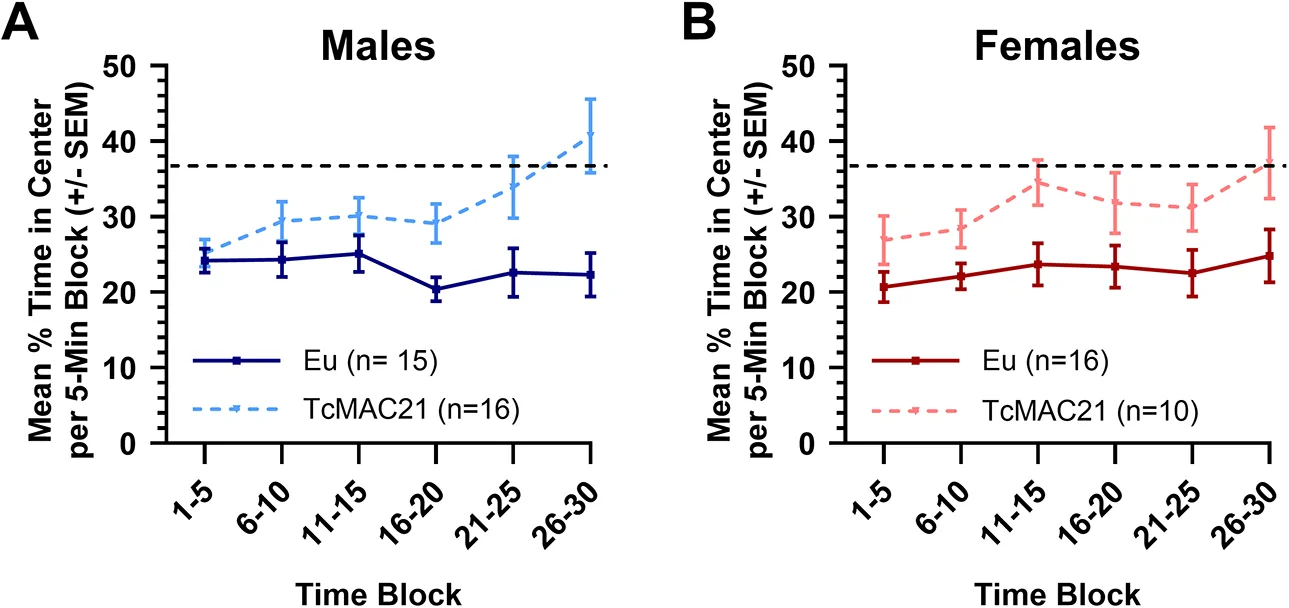
**Percentage time-in-center data shown in blocks of 5 min.** (A,B) The time-in-center data from the locomotor activity testing shown in Fig. 2 are replotted here as percentage of time over blocks of 5 min (males, A; females, B). This facilitates comparison of the current study with that of Kazuki et al. (2020), who reported parallel effects of increased percentage time-in-center of TcMAC21 mice in their open-field test. Dashed lines represent the expected value of percentage time in the center for each time block if activity was distributed randomly between center and periphery, based on the proportional surface area of the two. Eu, euploid. Euploid, male (*n*=15); TcMAC21, male (*n*=16); euploid, female (*n*=16); TcMAC21, female (n=10).

### Gait analysis

Multivariate analysis of the three gait parameters (stance width, step angle and stride length) yielded a significant main effect of genotype [Wilks' λ=0.700, F(3,61)=8.716, *P*<0.001; η^2^_p_=0.300]. The genotype×sex interaction did not reach significance [Wilks' λ=0.884, F(3,61)=2.658, *P*=0.056; η^2^_p_=0.116], nor did the main effect of sex [Wilks' λ=0.923, F(3,61)=1.689, *P*=0.170; η^2^_p_=0.077]. Univariate analyses indicated that the only significant outcome was a main effect of genotype for stride length [Fig. 4C; F(1,63)=3.204, *P*<0.001; η^2^_p_=0.189], with TcMAC21 mice of both sexes having longer stride lengths than those of euploid mice (simple effect of genotype within each sex, *P*=0.002 for males, *P*=0.023 for females). No main or interactive effects of sex were present for stride length, and no significant effects of genotype or sex were found for stance width (Fig. 4A) or step angle (Fig. 4B). Males and females differed significantly in body length [Fig. 4D; main effect of sex, F(1,63)=27.395, *P*<0.001; η^2^_p_=0.303], with no significant main or interactive effects of genotype. An analysis of covariance (ANCOVA) of stride length with body length as a covariate also yielded a significant main effect of genotype [F(1,62)=3.588, *P*<0.001; η^2^_p_=0.217] due to the longer stride lengths of TcMAC21 mice, indicating that the genotype effects on stride length were not associated with differences in body length.

**Fig. 4.**
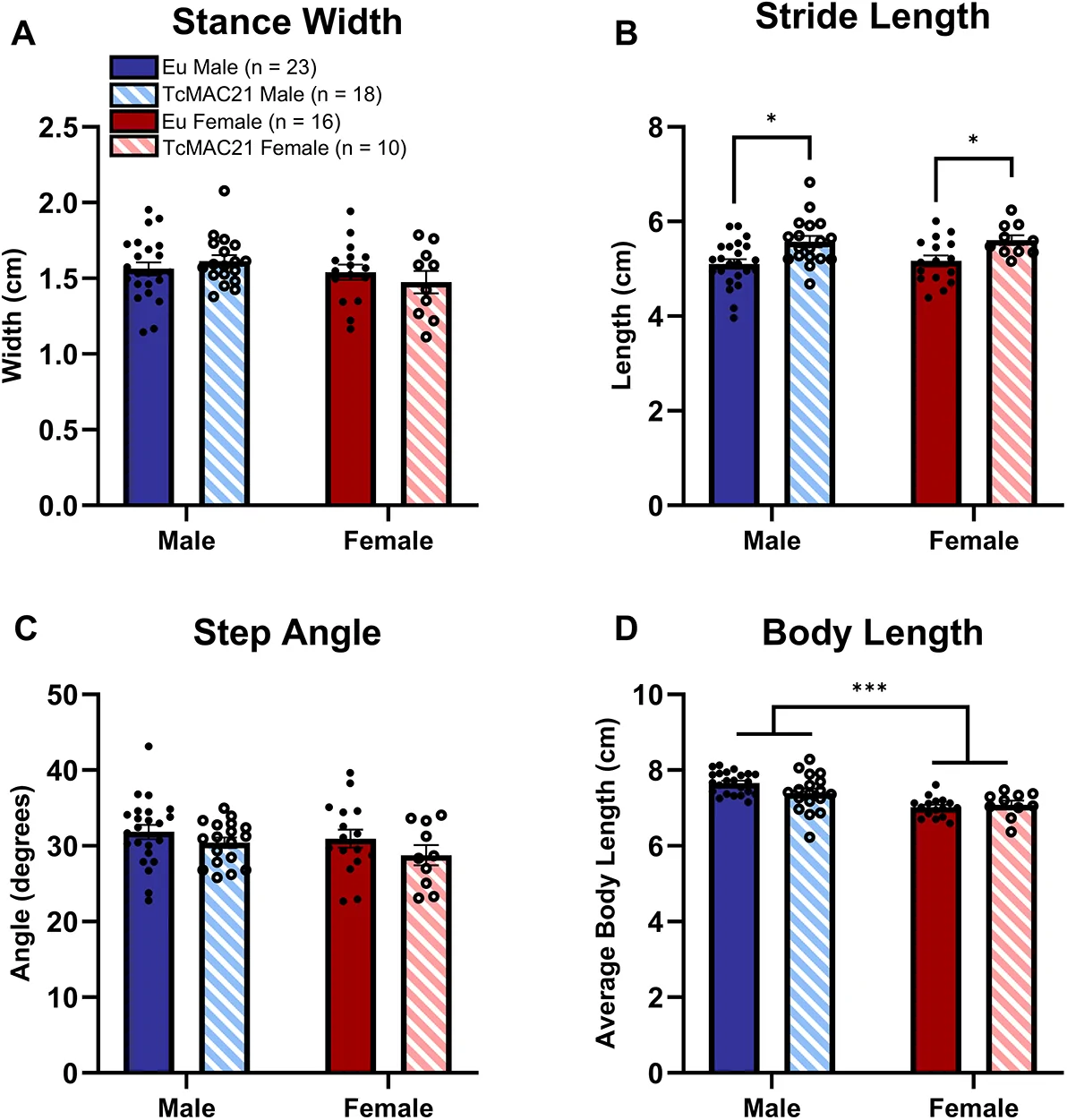
**Gait analysis of male and female TcMAC21 and euploid control mice.** (A,C) No significant effects of sex or genotype were found in analysis of stance width (A) or step angle (C). (B) A main effect of genotype was identified in stride length, wherein both male and female TcMAC21 animals had significantly longer strides than euploid littermates [main effect of genotype (*P*<0.001; η^2^_p_=0.189); *P*=0.002 for males; *P*=0.023 for females]. (D) Body length was significantly affected by sex (*P*<0.001; η^2^_p_=0.303) but not by genotype. *P<0.05; ***P<0.001; simple main effects. Euploid, male (*n*=23); TcMAC21, male (*n*=18); euploid, female (*n*=16); TcMAC21, female (*n*=10).

### Balance beam

No significant differences between the TcMAC21 and euploid mice were evident on the balance beam task (see Fig. S3), confirmed by the lack of main or interactive effects of genotype in the three-way ANOVA. Regardless of genotype, sex differences were evident as a function of beam width [sex×beam width interaction, F(1,63)=14.26, *P*<0.001; η^2^_p_=0.185], with male mice showing increased paw slips on the narrower beam 2 than on beam 1 (simple effects of beam width in males, *P*<0.001), whereas female mice showed no significant changes in paw slips due to beam width (*P*=0.875).

### Swim-escape adaptation

For the swim-escape adaptation training (Fig. S4), during the visible platform training (conducted first), all groups showed reduced latency to escape from the first to the second visible platform trials, then continued near-asymptotic performance on the two subsequent submerged platform trials. The mixed RM-ANOVA yielded significant effects of phase (*P*<0.001) and trial (*P*<0.001), and a phase×trial interaction (*P*<0.001). Follow-up RM-ANOVAs performed separately on the two phases confirmed significant reductions from trial 1 to trial 2 during visible training [main effect of trial, F(1,63)=38.60, *P*<0.001; η^2^_p_=0.380], along with a significant main effect of genotype [F(1,63)=4.539, *P*=0.037; η^2^_p_=0.067] due to the longer latencies of TcMAC21 mice. No other main or interactive effects were significant for visible training. For hidden platform training, there were no significant main or interactive effects of any factor, consistent with the data for all groups being near asymptote for escape performance on both trials. These data confirm that the swim adaptation training accomplished the goal of training the mice to comparable levels of swim escape competence in advance of the Morris water maze (MWM) training.

### Morris water maze

#### Visible platform training

During cued training, individual latency measures were strongly correlated with the respective path lengths for each trial (Pearson's *r* of 0.967, 0.920, 0.963, 0.905 and 0.849 for the five trials, respectively; *P*<0.01 for all), and the two measures yielded comparable outcomes. There were no significant differences between TcMAC21 and euploid mice in latency (Fig. 5) or path length (Fig. S5) to reach the platform on the five visible platform trials, nor were there any main or interactive effects of sex. Over the five trials, all groups showed significantly reduced latency [main effect of trial, F(2.692,169.591)=17.32, *P*<0.001; η^2^_p_=0.216] and path length [main effect of trial, F(2,758)=173.744, *P*<0.001; η^2^_p_=0.210], and data were asymptote by the fifth trial. For the additional measures of MWM performance during cued training (Fig. S6), the average swimming speed of TcMAC21 mice was 11.3% lower than that of euploid mice [main effect of genotype, F(1,63)=5.302, *P*=0.025; η^2^_p_=0.078], with overall means (sexes combined) of 0.228±0.009 m/s versus 0.257±0.009 m/s, respectively. There were no significant effects of genotype or sex on percentage time floating or on heading angle (Table S1).

**Fig. 5.**
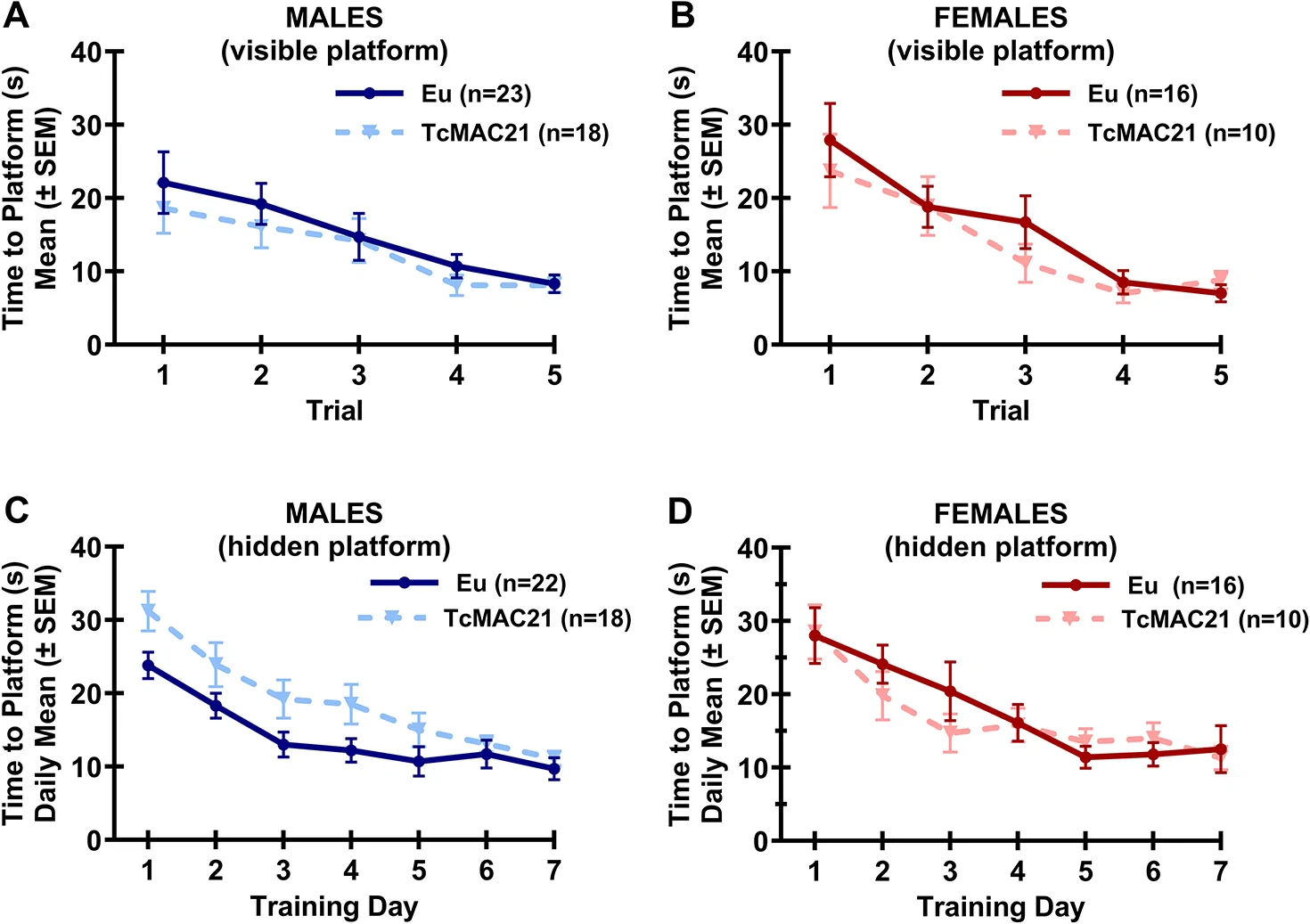
**Mean latency to the platform of male and female TcMAC21 and euploid littermate mice during cued training and place training.** (A,B) Cued training on the visible platform task shows the mean latency for each trial in males (A) and females (B). (C,D) For place training on the hidden platform task in males (C) and females (D), latencies were averaged for the four daily trials each day for each individual, and the average of the daily means are shown for the 7 days. Performance on cued training was not significantly affected by genotype or sex, and all groups showed significant reductions in latencies over trials (*P*<0.001 for all). For place training, all groups showed significant reductions in latency to the platform over days (*P*<0.001 for all), with no significant main or interactive effects of genotype or sex. Error bars represent s.e.m. Euploid, male (*n*=23); TcMAC21, male (*n*=18); euploid, female (*n*=16); TcMAC21, female (*n*=10).

#### Hidden platform training

For acquisition of place learning, correlations between the individual average daily latencies to reach the platform and the average daily path lengths were consistently strong for each of the 7 days of training (Pearson *r*>0.936 for all, *P*<0.01 for all). There were no significant differences between TcMAC21 and euploid mice for either measure of place acquisition learning on the three-way RM-ANOVAs. All groups showed significant reductions over days in latency to reach the hidden platform [Fig. 5C,D; main effect of day, F(5.089, 315.655)=35.531, *P*<0.001; η^2^_p_=0.364] and in path lengths [Fig. S5; main effect of day, F(4.976,308.531)=42.153, *P*<0.001; η^2^_p_=0.405]. For latencies, there were no significant main effects of genotype [F(1,62)=1.320, *P*=0.255; η^2^_p_=0.021] or sex [F(1,62)=0.220, *P*=0.641; η^2^_p_=0.004], the genotype×sex interaction was not significant [F(1,62)=2.995, *P*=0.089; η^2^_p_=0.046], and there were no significant interactions with day. Similarly for path length, there were no significant main effects of genotype [F(1,62)=0.204, *P*=0.653; η^2^_p_=0.003] or sex [F(1,62)=0.293, *P*=0.590; η^2^_p_=0.005], the genotype×sex interaction was not significant [F(1,62)=3.120, *P*=0.082; η^2^_p_=0.048], and there were no significant interactions with day. Two additional measures of MWM performance during place training are shown in Fig. S6. For swimming speed, the main effect of genotype for swim speed was significant [F(1,62)=10.98, *P*=0.002; η^2^_p_=0.150], but no main or interactive effects of sex were significant. The speeds of TcMAC21 mice (mean=0.2205± 0.0051 m/s, sexes combined) were 11.6% lower than those of euploid mice (mean=0.2495±0.0061 m/s, sexes combined). For percentage time floating, thigmotaxis (Table S1) and absolute heading angle (Table S1), there were no significant main or interactive effects of genotype or sex.

#### Probe trial

The primary measure of the strength of an acquired place memory – the percentage time in the four virtual counters during the probe trial conducted at the start of the sixth day of acquisition training – is shown in Fig. 6. All groups displayed a significant spatial bias toward the location in the target quadrant compared to the matched locations in the other three quadrants, yielding only a significant main effect of quadrant [F(1.864,113.726)=91.53, *P*<0.001; η^2^_p_=0.600] in the overall three-way RM-ANOVA. There were no significant main or interactive effects of genotype or sex (*P*>0.15 for all). For all groups, the percentage time in the target counter was approximately four times greater than chance, whereas the percentage time in non-target counters did not exceed chance. Similar place biases were observed for both sexes for the number of passes through the target quadrant compared to non-target quadrants (Fig. S8). The strong spatial bias shown in the probe trial data confirms that all groups had acquired a spatial memory for the location of the platform after 5 days of acquisition training.

**Fig. 6.**
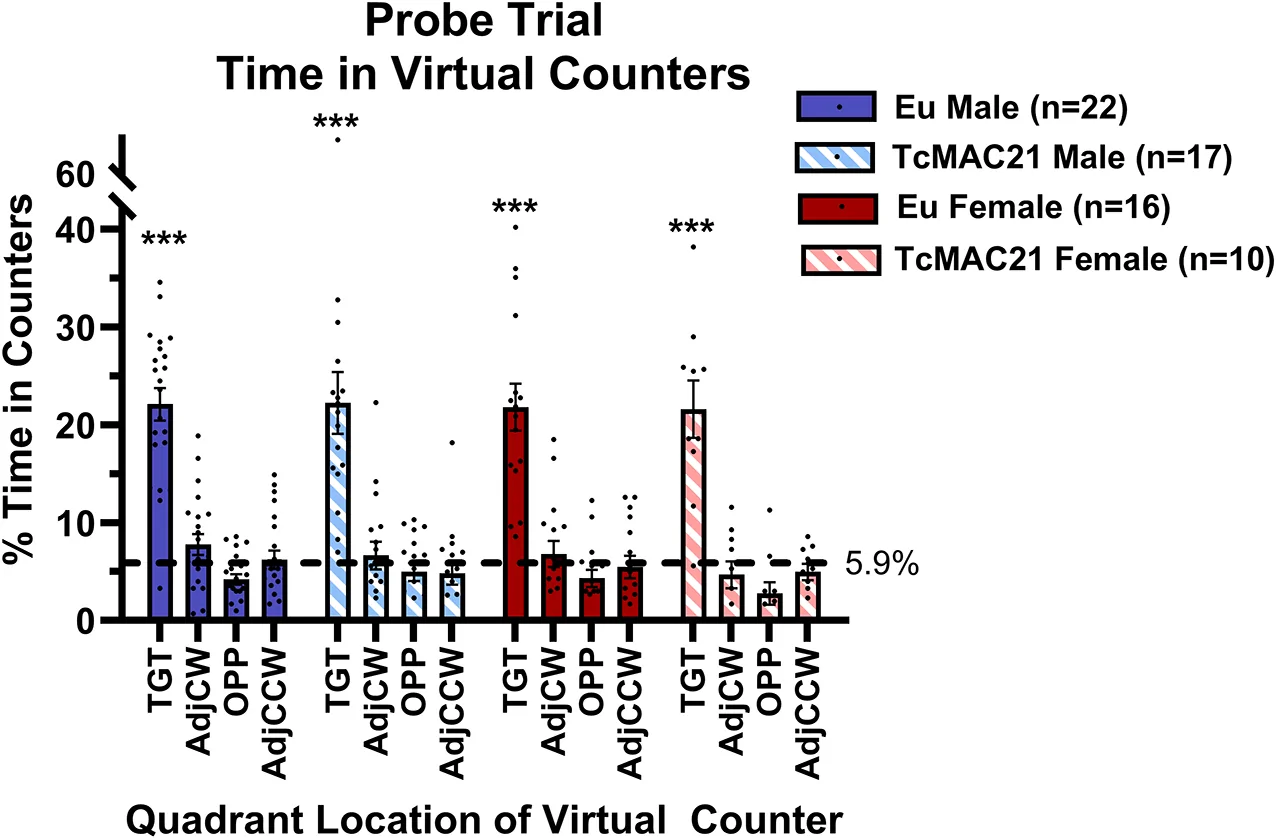
**Percentage time spent in each of the four virtual counters during the probe trial.** All groups spent significantly more time in the counter in the target quadrant (TGT) than in the counters in the three non-target quadrants (AdjCW, adjacent clockwise; OPP, opposite; AdjCCW, adjacent counterclockwise). There were no significant main or interactive effects of genotype or sex. The dashed line shows the expected percentage time in each counter for a randomly distributed search (5.9%). ***P<0.001, paired comparisons of percentage time in target compared to that in each non-target location within each group (using Bonferroni corrections). Euploid, male (*n*=22); TcMAC21, male (*n*=17); euploid, female (*n*=16); TcMAC21, female (*n*=10).

During the probe trial, TcMAC21 mice swam significantly shorter distances [F(1,61)=5.509, *P*=0.022; η^2^_p_=0.83] and had significantly slower swim speeds [reduced by 7.1%; F(1,61)=4.834, *P*=0.032; η^2^_p_=0.73] than those of euploid mice (Fig. 7). There were no main or interactive effects of sex on distance or swim speed (*P*>0.67 for all). For float time, heading angle or percentage thigmotaxis time, there were no significant main or interactive effects of genotype. One notable sex difference was present in both genotypes: females showed significantly more thigmotaxis during the probe than did their male counterparts [F(1,61)=8.941, *P*=0.004; η^2^_p_=0.128].

**Fig. 7.**
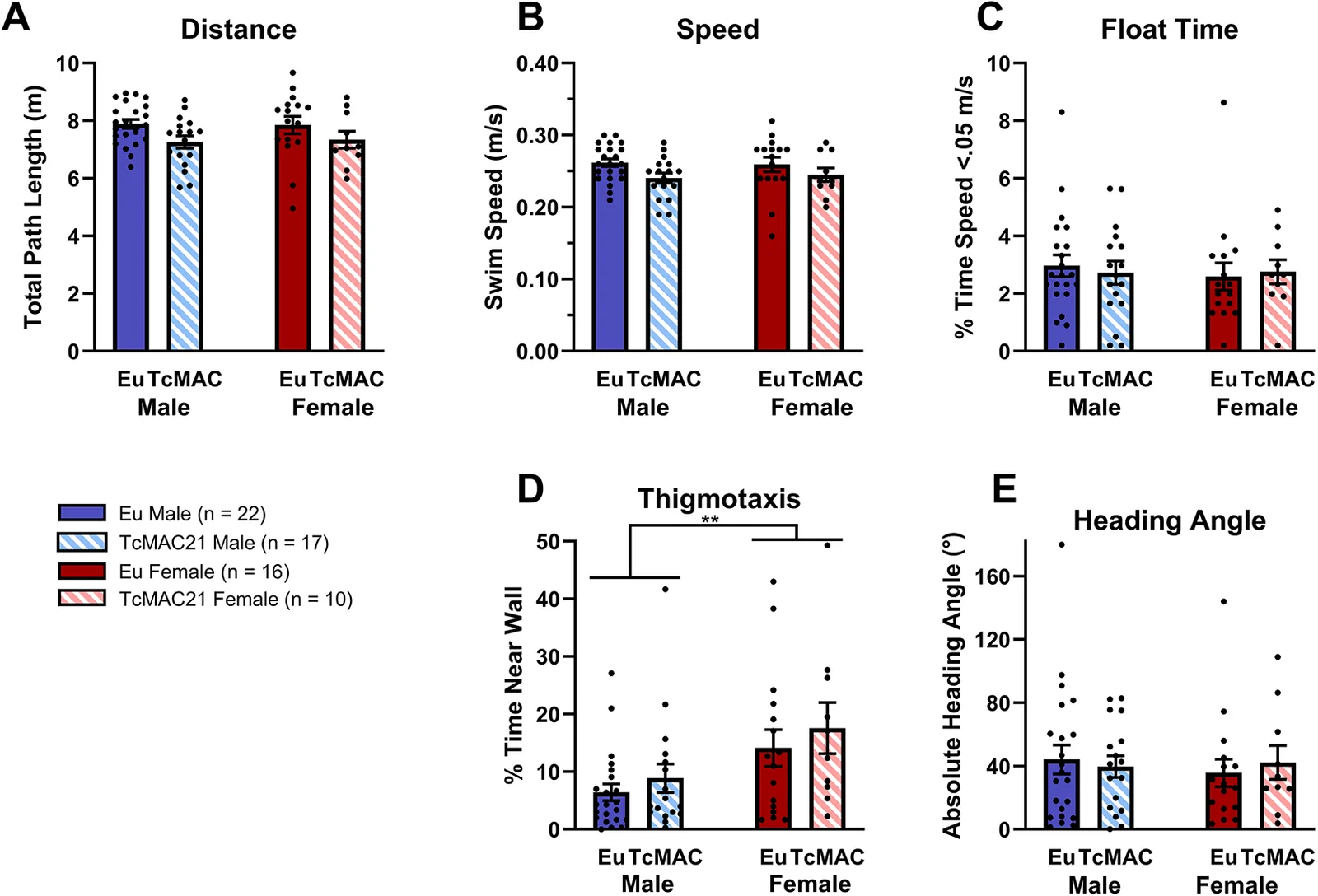
**Probe trial path length, swimming speed, float time, thigmotaxis, and heading angle of male and female TcMAC21 and euploid littermate mice.** (A,B) TcMAC21 mice swam shorter distances (A) and had slower swim speeds (B) than euploid mice (main effects of genotype, *P*=0.022 and *P*=0.032, respectively). (C) Float time was not significantly different between male or female TcMAC21 and euploid littermate mice. (D) For thigmotactic behaviors of mice, sex differences were present, with female mice spending a significantly increased percentage of time near the walls of the maze. (E) Heading angle was not significantly different between male or female TcMAC21 and euploid littermate mice. **P<0.01, simple main effects. Euploid, male (*n*=22); TcMAC21, male (*n*=17); euploid, female (*n*=16); TcMAC21, female (*n*=10).

#### Reversal training

On the day after the seventh day of place training, reversal training was implemented in which the hidden platform was moved to the quadrant opposite the one used during acquisition, and it remained in that position for 3 consecutive training days. The latencies to reach the platform were, again, highly correlated with path lengths across the individual reversal trials (*r*>0.925 for all). As shown in Fig. 8 (latencies), all groups displayed the expected effect of the reversal contingency, with long latencies on the first reversal trial that declined significantly over the first day, with further reductions over the second day. Asymptotic performance was achieved by the third day [main effect of day, F(2,116)=65.50, *P*<0.001; η^2^_p_=0.530; main effect of trial, F(3,174)=29.42, *P*<0.001; η^2^_p_=0.337; day×trial interaction, F(4.997,289.854)=11.245, *P*<0.001; η^2^_p_=0.162]. Unexpectedly, significant effects of genotype were evident only in male mice, confirmed by a significant genotype×sex interaction [F(1,58)=5.940, *P*=0.018; η^2^_p_=0.093]. Comparable outcomes were present in path lengths (Fig. S7), including significant reductions over training [main effect of day, F(1.804,104.61)=62,51, *P*<0.001; η^2^_p_=0.519; main effect of trial, F(3,174)=38.488, *P*<0.001; η^2^_p_=0.399; day×trial interaction, F(4.827,279.99)=10.519, *P*<0.001; η^2^_p_=0.154] and significant genotype×sex interaction [F(1,58)=4.818, *P*=0.032; η^2^_p_=0.077].

**Fig. 8.**
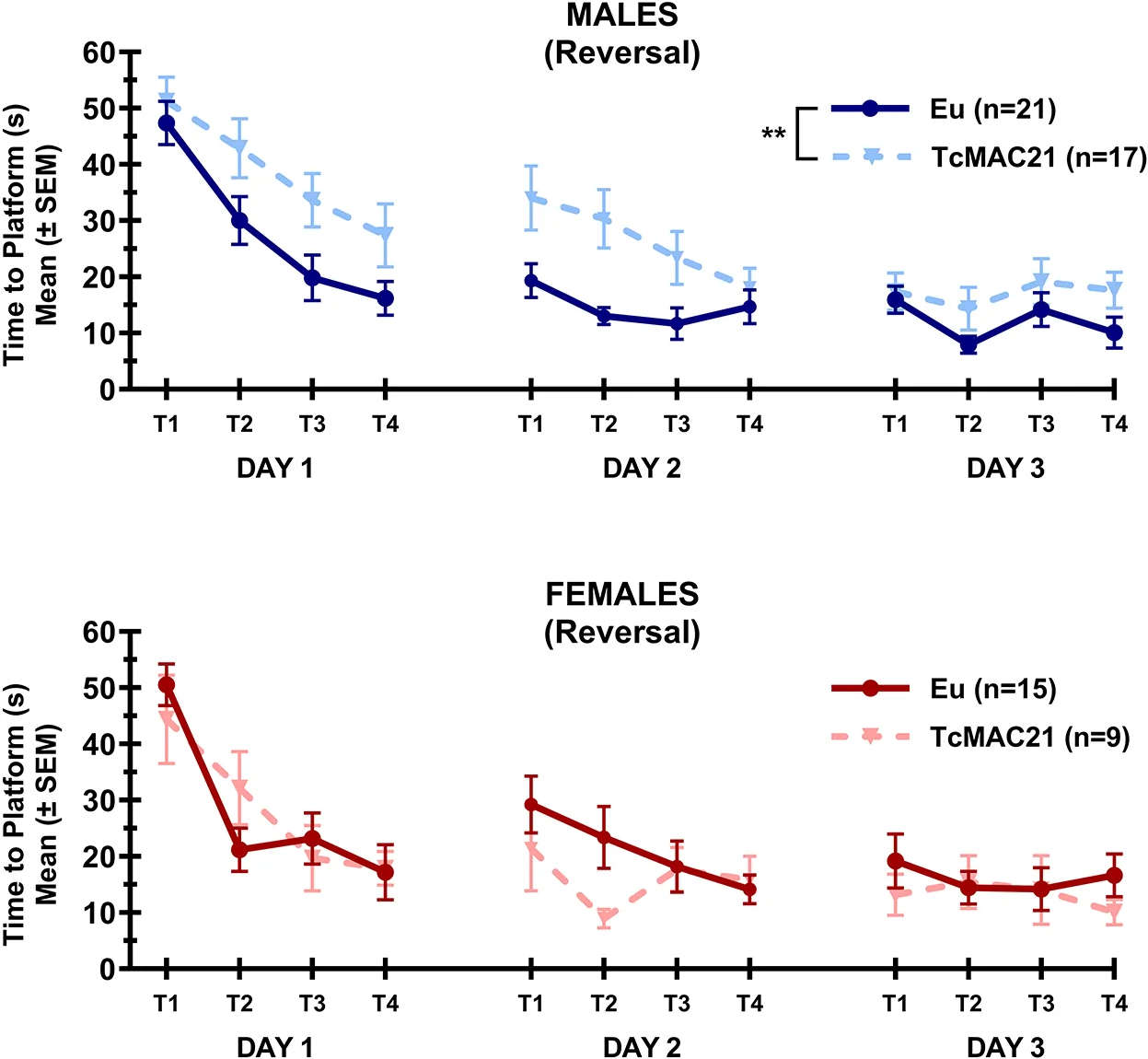
**Latency to reach the platform across 3 days of reversal training.** Male TcMAC21 mice, but not female TcMAC21 mice, showed significant impairments in learning the reversal contingency (genotype×sex interaction, *P*=0.018). Follow-up analyses within each sex confirmed that male TcMAC21 mice had longer latencies during reversal than did male euploid littermates (main effect of genotype, **P=0.006). No significant genotype differences in latencies to reach the platform were present in females (*P*=0.455). Euploid, male (*n*=21); TcMAC21, male (*n*=17); euploid, female (*n*=15); TcMAC21, female (*n*=9).

The genotype×sex interactions were followed up by separate RM-ANOVAs for each sex. TcMAC21 male mice had significant deficits in learning the reversal contingency compared to euploid males, with slower learning curves marked by significantly longer latencies (Fig. 8, main effect of genotype, *P*=0.006; η^2^_p_=0.192) and path lengths (Fig. S7, *P*=0.020; η^2^_p_=0.141) to locate the platform across trials, most prominently on the first 2 days of reversal. The reversal learning deficits in males contrasted sharply with the absence of deficits in female TcMAC21 mice (Fig. 8).

The TcMAC21 mice also showed significantly slower swimming speeds during reversal training (Fig. S6), with overall average speeds that were 10.4% lower than those of euploid mice [main effect of genotype, F(1,58)=4.54, *P*=0.037; η^2^_p_=0.073]. These reductions were comparable to the slower swimming speeds of TcMAC21 in all other training phases (visible, hidden and probe). The slower swimming speeds throughout MWM testing could not account for sex-specific deficits evident only in reversal performance in TcMAC21 mice, given the absence of significant differences between males and females on swimming speed during reversal training (*P*>0.29 for all). Additionally, the slower swimming speeds in other training phases were not associated with any group differences in measures of learning or memory during visible, hidden or probe trials. No differences in float time, thigmotaxis or heading angle were seen in males during reversal training.

## DISCUSSION

The potential translational value of the transchromosomic TcMAC21 humanized mouse model of DS has been acknowledged since the initial characterization showed that the model contained ∼93% of Hsa21q protein-coding genes and recapitulated multiple DS phenotypes (Kazuki et al., 2020). In tests of cognitive and behavioral phenotypes in 3-month-old mice, that study reported that TcMAC21 mice had reduced display of anxiety-like behavior in a novel open field (without differences in overall locomotor activity), and deficits in place learning and memory in a MWM place acquisition task (both sexes tested) and in repeated reversal tests (only females were tested). The only other report, to date, on TcMAC21 mice that addressed DS-related effects on learning showed significant deficits in complex motor learning in the Erasmus maze and in accelerated rotarod performance, which were associated with cerebellar structural deficits and functional impairments in cerebellar circuitry (Stander et al., 2026). The current study adds to this limited experimental literature by assessing behavioral and cognitive phenotypes in male and female TcMAC21 mice during late adolescence and young adulthood (P45-P62). Key findings included growth deficits (more pronounced in males), reduced expression of anxiety-related behavior in a novel arena (replicating Kazuki et al., 2020), altered gait and, most notably, sex differences in the effects on MWM performance, in which males, but not females, showed prominent deficits in reversal of place learning. This male-specific impairment in MWM reversal learning in TcMAC21 mice is the first demonstration of sex-dependent deficits in cognitive flexibility in mouse models of DS.

Cognitive flexibility is a component of executive function that refers to the ability to change responses under changing environmental demands, including the ability to switch between rules and strategies when outcome probabilities change, and is an essential trait in adaptive behavior (Brown and Tait, 2010). The current study adds to converging evidence from DS mouse models and individuals with DS that indicates that diminished cognitive flexibility is a significant developmental phenotype in DS and supports expanded translational analysis of cognitive flexibility in mouse models of DS.

The deficits in MWM reversal learning in male TcMAC21 mice at ∼2 months of age are consistent with the previous finding of impaired cognitive flexibility in male Ts65Dn mice at ∼2 months of age (Olmos-Serrano et al., 2016). That study provided a longitudinal analysis of cognitive flexibility in Ts65Dn mice (males only) using spatial tasks that required learning to reverse previously acquired place discrimination learning either in a water T-maze or in a standard MWM. They concluded that “diminished cognitive flexibility, as measured by reversal learning, is the most robust learning and memory impairment in both young and old Ts65Dn mice” (Olmos-Serrano et al., 2016). Similar work in the Dp16 mouse model (on a B6 background, compared to TcMAC21 and Ts65Dn mice on a B6C3 advanced intercross background) at ∼3 months of age (Goodliffe et al., 2016) is fully consistent with that conclusion, yet both studies tested only males, leaving open the question of whether deficits in cognitive flexibility also apply to females in mouse models of DS.

The current study extends the previous findings in Ts65Dn and Dp16 mouse models to TcMAC21 mice and provides the first demonstration of sex-specific cognitive flexibility impairments in mouse models of DS. The deficits in males, but not females, were confirmed by the significant genotype×sex interactions (with medium effect sizes) in both the latency and the path length measures on reversal performance. Kazuki et al. (2020) reported a transient, but significant, MWM reversal deficit in female TcMAC21 mice, suggesting that female TcMAC21 mice display cognitive flexibility impairments under certain testing conditions. However, there were major differences between the MWM training protocols of Kazuki et al. (2020) and the current study (discussed below) that may have produced differences in the acquisition and reversal performance between the two studies. More pertinently, Kazuki et al. (2020) did not test males in the repeated reversal protocol, leaving unaddressed whether sex differences would have been present under their training conditions and, if so, whether males would have larger deficits.

The extent to which the hypermetabolism phenotype previously reported in the TcMAC21 model (Sarver et al., 2023) contributes to impaired cognitive flexibility in this model, and whether and how it may differentially affect male and female animals, is unknown. Impaired executive function and cognitive flexibility are associated with a variety of metabolic disorders, including types 1 and 2 diabetes and metabolic syndrome, especially at later ages (Yermakov et al., 2019; Nooyens et al., 2010; Haase Alasantro et al., 2021; Mojun et al., 2025), but the extent to which these may shed light on cognitive function profiles in the hypermetabolic TcMAC21 model is uncertain.

Impairments in cognitive flexibility and executive function reported in individuals with DS (Campbell et al., 2013; Heyman and Hauser-Cram, 2015; Phillips et al., 2014; Zelazo et al., 1996) have received increased scrutiny in efforts to advance psychometric standards to produce reliable and valid measures for individuals with DS (Schworer et al., 2023). The current findings in the TcMAC21 mouse model implicate a previously unrecognized sex difference in cognitive flexibility impairments that may be evident in young adulthood, perhaps recapitulating timelines in which phenotypes extend for longer periods of development in males than in females with DS (Baumer et al., 2024). Limited existing research of sex differences in cognitive ability in people with DS has suggested a role of sex on intellectual quotient (IQ) and verbal cognition (Del Hoyo Soriano et al., 2024), and sex differences in executive function may emerge during development (Edgin et al., 2010; Miezah et al., 2026). The findings of mouse model studies indicate that diminished cognitive flexibility is a significant developmental phenotype in DS and supports expanded translational analysis of cognitive flexibility in DS mouse models. Translational studies involving reversal learning or attentional set-shifting tasks are feasible using operant procedures (Birrell and Brown, 2000; Bissonette and Powell, 2012; Garner et al., 2006; Tait et al., 2014; Ciampoli et al., 2021) – including ones incorporating touch-screen responses (Mar et al., 2013; Martins et al., 2025) and ones with demonstrated sensitivity to sex differences (Moench et al., 2020). These provide opportunities to model comparable tests used in humans such as the Wisconsin Card Sorting task involving intra-dimensional and extra-dimensional shifts and reversal learning (Highgate and Schenk, 2021). Aligning studies of cognitive flexibility in mouse models and individuals with DS could expand the prospects for translational approaches in DS research to advance and improve understanding of the mechanisms underlying and interventions to improve cognitive phenotypes.

Multiple neural systems are known to contribute to cognitive flexibility and, more generally, to executive functioning, with the prefrontal cortex and associated thalamic circuits being primarily implicated and conserved across species (Sakai, 2008; Li et al., 2017; Floresco et al., 2008; Chudasama, 2011; Friedman and Robbins, 2022; Der-Avakian et al., 2025), along with cerebellar-forebrain circuits (Dickson et al., 2010) and hippocampal-cortical circuits (Rubin et al., 2014), all of which have been implicated in DS (Lott and Dierssen, 2010; Pennington et al., 2003; Russo et al., 2024). Cortico-cerebellar circuits may be particularly involved in deficits in cognitive flexibility in DS. Cerebellar hypoplasia and functional impairments in cerebellar circuitry constitute a primary neurological phenotype in mouse models of DS, with strong predictive validity for translational studies of DS (Baxter et al., 2000). The cerebellum is recognized to have a role in cognitive processing, and cerebellar structural deficits and impaired function of cerebro-cerebellar circuitry have been linked to impaired cognitive processes (Desmond and Fiez, 1998; Koziol et al., 2014), including deficits in cognitive flexibility and executive control when previously learned contingencies change, as during reversal learning or set-shifting tasks (Dickson et al., 2010; Rogers et al., 2011; Goodlett and Mittleman, 2017). Taken together, these considerations support studies in DS mouse models designed to assess cerebellar regulation of cognitive flexibility.

The lack of significant differences between TcMAC21 and euploid mice in acquisition of place learning contrasts with the findings of Kazuki et al. (2020), who showed significant deficits in acquisition of MWM place learning in males but not females. However, there were major differences in the MWM acquisition training protocols between the two studies. The present study used a typical MWM place learning acquisition protocol with a fixed platform (four trials per day, 7 consecutive days), with a single probe trial with no platform present (in this case, as the first trial of day 6). Kazuki et al. (2020) used an acquisition protocol that involved eight place training trials per day for 4 consecutive days, with probe trials (with a collapsible platform temporarily unavailable for 30-40 s then raised to provide escape) interposed each day, one 30 min after the last of eight daily place training trials, and a second 24 h later as the first trial of the next day. Having six interposed probe trials during acquisition introduces repeated episodes of potential extinction/re-acquisition effects that may both slow the rate of acquisition (compared to typical training) and change the type of place learning from one of basic spatial navigation to one incorporating additional elements of learned persistence with more localized search during the ‘dwell’ times when the platform is unavailable. The significant deficits in MWM acquisition found by Kazuki et al. (2020) in TcMAC21 males, but not females, contrast with the lack of significant acquisition deficits in either sex in the current study and may reflect differences in acquisition performance resulting from the two training procedures used. Perhaps of more direct relevance, deficits in MWM acquisition of place learning have been commonly reported in previous DS mouse model studies under various training protocols; most of those used Ts65Dn mice (including those from this laboratory), although acquisition deficits have also been reported for Dp16 mice (Rueda et al., 2012; Herault et al., 2017; Reeves et al., 1995; Goodliffe et al., 2016; Olmos-Serrano et al., 2016; Stringer et al., 2017, 2015; Goodlett et al., 2020; Yu et al., 2010). Most of the MWM studies on DS mouse models evaluated only males; the few that included both sexes either pooled the data or were underpowered to detect sex differences (or both). Notably, the extent of place acquisition deficits has been correlated with the amount of thigmotaxis expressed in the early stages of training (Olmos-Serrano et al., 2016; Stasko and Costa, 2004; Vorhees and Williams, 2014). Our initial swim adaptation procedure combined with initial training with the visible platform MWM task may account for the lack of group differences in thigmotaxis behavior in the MWM place task.

In traditional open-field tests, rodents typically show thigmotaxic behavior in novel arenas, spending a disproportionate amount of time in the periphery (near the walls) compared to in the center of the arena. That thigmotaxic behavior associated with avoidance of the center of the arena is usually interpreted as a form of anxiety-like behavior (often linked ecologically in rodents to potential exposure to predatory threats) that is a consequence of anxiety evoked when encountering the novel open-field arena (Kraeuter et al., 2019; Prut and Belzung, 2003; Seibenhener and Wooten, 2015; Walsh and Cummins, 1976; Treit and Fundytus, 1988), although such effects may be limited or insufficiently validated (Stanford, 2007; Thompson et al., 2015). The species-typical thigmotaxis response stands in contrast to the reduced anxiety-like behavior observed in the TcMAC21 model, in which mice of both sexes spent significantly more time in the center of the arena than did controls, an effect that became more pronounced in the second half of the session. These activity tests were conducted in enclosed activity chambers in the dark, thereby limiting several key features of traditional open-field tests that can enhance the expression of thigmotaxis, including illumination level and open exposure to the external environment (Walsh and Cummins, 1976; Post et al., 2011). Nevertheless, our results largely replicated the outcomes reported by Kazuki et al. (2020), who used a traditional open-field test (compare our Fig. 3 with fig. 6d of Kazuki et al., 2020; both shown in the same format with percentage time-in-center plotted in 5-min intervals). These similar outcomes were evident despite the major methodological differences, including age of testing (P45 versus 3 months for Kazuki et al., 2020), along with the testing conditions noted above. In our study, activity was assessed in a novel 40.6 cm×40.6 cm arena (fitted with arrays of infrared photocell detectors) that was enclosed in a light-tight, sound-attenuating environmental chamber, tested in the dark during the dark phase of the circadian period. Kazuki et al. (2020) assessed activity in a traditional open-field test in a 37 cm×37 cm arena open to the room that was illuminated by indirect diffuse light (150 lux) using video tracking software (circadian phase not specified). Despite these methodological differences, the time-in-center measures show comparable effects of genotype across the session. A similar outcome for time-in-center was also observed in male mice of the transchromosomic Tc1 mouse model (Galante et al., 2009), although this model has several genomic alterations in the extra chromosome and mosaicism (Gribble et al., 2013). Note that, in our test, over the second half of the session, the amount of time spent in the center approximated what would be achieved if it had been randomly distributed across the arena (chance indicated by the dashed line in Fig. 2B,D,F). One implication is that the exploration of TcMAC21 mice in the novel arena appears to be unaffected by environmental features that typically evoke anxiety (that drives thigmotaxis) and may instead reflect an expression of motor activity patterns that are less influenced by input from neural systems involved with processing emotional valence.

Findings from open-field and activity studies in the literature on anxiety-like behavior for DS mouse models are mixed. Adult Ts65Dn mice were reported to show less species-typical thigmotaxis than euploid controls in the open field (Coussons-Read and Crnic, 1996), and some studies (Demas et al., 1996; Coussons-Read and Crnic, 1996) reported that trisomic mice showed significantly fewer anxiety-like phenotypes that are typically expressed in the elevated plus maze. Others have reported an increase in anxiety-like behavior in Ts65Dn mice (Llambrich et al., 2024) and in the Dp(10)2Yey model (Muza et al., 2023). Anxiety states in situations with potential threat can evoke behaviors that serve protective or adaptive roles, and reduced expression of species-typical anxiety traits would be detrimental in those situations. As the TcMAC21 model shows decreased anxiety-like behavior across multiple tasks that assess species-typical anxiety, this model may be useful for identifying neural circuitry and mechanisms underlying the differences in motivational and emotional components of anxiety traits in DS.

The current study is the first to use balance beam traversal to evaluate motor performance in TcMAC21 mice. Traversal deficits were evident at narrow beam widths in a previous study in Ts65Dn mice (Stringer et al., 2015); however, deficits were not observed in a different study using Ts65Dn mice with a different developmental history (Goodlett et al., 2020). The lack of effects in the TcMAC21 mice in the current study was not due to any lack of sensitivity to the increased difficulty at narrower beam widths, given that males (but not females) displayed the expected increase in frequencies of hind paw slips at the narrower beam. For both genotypes, males were significantly larger (greater body weight and longer body length) than females, and that larger size may increase the likelihood of hind paw slips on the narrower beam. Nevertheless, it appears that the balance beam task, which relies on a fundamental component of hindlimb stepping, may be less sensitive to motor learning deficits in DS mouse models than more complex or dynamic tests of motor coordination and motor learning that place greater demands on cerebellar function, such as the Erasmus Ladder (Stander et al., 2026).

The separate analysis of gait showed that both male and female TcMAC21 mice had significantly longer stride lengths than those of controls, even when compared to total body length as a ratio. That alteration of gait may result from multiple sources, including cerebellar regulation or skeletal or musculature development. Previous data from individuals with DS and from DS mouse models have identified alterations in gait, typically as a reduced step distance and wider step angle and width (Scott-McKean et al., 2023; Zago et al., 2020). The discrepancy between the current findings and previous findings in other DS mouse models may point to differences in either genetic or developmental processes in the acquisition of motor control in the TcMAC21 model versus other models of DS.

Slower growth and delayed development are commonly observed in individuals with DS (Hatch-Stein et al., 2016). The current study confirmed significantly lower body mass and slower growth from P45 to P62 in male and female TcMAC21 mice compared to that in sex-specific littermates, but with increasingly larger differences in males than in females. These were consistent with the initial report on the TcMAC21 model (Kazuki et al., 2020). The slower growth trajectory of TcMAC21 that is exacerbated in males highlights the importance of analyzing both male and female mice with sufficient power to detect potential sex differences. Given that TcMAC21 mice show hypermetabolism compared to euploid controls, the reductions in body weight and size in TcMAC21 animals may be associated with the increased metabolic rate (Faizi et al., 2011; Sarver et al., 2023). Furthermore, the unique chronic hypermetabolic state in the TcMAC21 model may have different or sex-specific effects across many domains of function, including cognition, motivation, behavioral state regulation and sleep, or neuronal and brain functioning.

### Conclusions and implications

This study replicated and extended findings from other studies on the TcMAC21 mouse model of DS (Kazuki et al., 2020; Stander et al., 2026; Kurabayashi et al., 2023), and provided evidence of the recapitulation of several DS-relevant phenotypes, including growth deficits, reduced anxiety-like behavior in a novel environment and sex-specific impairment in cognitive flexibility. Other phenotypes showed effects that were either limited (locomotor activity and gait) or not evident (balance beam performance). Cognitive flexibility mediates the ability to adapt behaviorally when environmental conditions or internal cues change – by switching responses, strategies or attentional set as conditions change – and is a key component of effective executive function. Core features develop rapidly during preschool years, yet protracted development continues through childhood, adolescence and into young adulthood (Buttelmann and Karbach, 2017). Its emergence is closely tied to family communication patterns, social function, personal wellbeing and achievement of important life outcomes, and it may be improved by metacognitive training (Buttelmann and Karbach, 2017; Kalkut et al., 2009; Koesten et al., 2009; Somsen, 2007). Given its developmental importance and the demonstrated deficits in DS, cognitive flexibility may be a phenotype that is especially well suited for experimental studies of DS mouse models with bidirectional translation to studies of individuals with DS, particularly when the two are designed in parallel. Translational validity across species appears relatively high when using paradigms such as reversal learning and attentional set shifting. Experimental approaches can be aligned between species, including in a developmental framework, and the underlying functional circuitry appears to be conserved across species (Der-Avakian et al., 2025; Heisler et al., 2015; Highgate and Schenk, 2021; Martins et al., 2025). Translational validity may be demonstrated (including the possibility of sex differences) when the conditions under which behavioral features of impaired cognitive flexibility seen in DS mouse models recapitulate or predict comparable deficits in individuals with DS. These may then provide avenues to identify correlated functional changes in underlying neural systems, along with ways to test behavioral or pharmacological interventions. This model provides a novel resource to examine the effects of Hsa21 genes in a murine model and may help to further elucidate the functions of these genes and the mechanisms from genes to cognitive phenotypes associated with DS.

## MATERIALS AND METHODS

### Mouse husbandry

Female Tc(HSA21,CAG-EGFP)1Yakaz/J (TcMAC21; Strain #035561) (Kazuki et al., 2020) and male B6D2F1 (Strain #100006) mice were purchased from The Jackson Laboratory (Bar Harbor, ME, USA). (TcMAC21×B6D2F1) mice were bred and maintained in colony rooms kept at 22±3°C with humidity ranging between 30% and 60% with a standard 12:12 h light:dark cycle (lights on at 07:00) in cages with sterile wood shaving bedding (Sani-Chips, Cincinnati Lab Supply) and pulped cotton fiber nestlets for enrichment (NES3600, Ancare). Mice were housed with access to food (Laboratory Rodent Diet 5001, LabDiet) and water *ad libitum*. New TcMAC21 and B6D2F1 mice were added to the colony breeding approximately every 6-12 months to minimize genetic drift. Mice were genotyped by PCR using transgene primers (5′-TTCAACCTGAGCTCCACATC-3′ and 5′-ATTACATCCCTGGGGGCTTT-3′) and internal positive control primers (5′-AGTGGCCTCTTCCAGAAATG-3′ and 5′-TGCGACTGTGTCTGATTTCC-3′), as recommended by The Jackson Laboratory (Strain #035561, Protocol 40719, Version 1.0). Whole litters were taken for testing unless female mice were needed for breeding and colony maintenance. Euploid controls were sex-matched littermates of the TcMAC21 animals. The lower numbers of female TcMAC21 mice in the groups undergoing behavioral testing was due to some females being taken periodically to serve as breeders.

Mice were weaned between P21 and P24, at which point they were transferred to a reversed light cycle room (12:12 h light:dark cycle; lights off at 07:00), with up to five same-sex animals housed per cage. Up to four animals were tested in a single cohort. A minimum of ten animals were tested per group (male, female, euploid, TcMAC21), which in previous studies yielded sufficient power to detect potential sex differences in bone and brain phenotypes (American Academy of Pediatrics, 2001; Hawley et al., 2024; Sloan et al., 2025 preprint). Starting on ∼P38, mice were individually handled by an experimenter for 1 min per day. Behavioral testing began on P45 and was conducted during the dark phase between 12:00 and 16:00 in testing rooms illuminated with either red light or indirect dim white light from LED bulbs. Body weights of mice were collected throughout testing, to monitor growth. Additional qualitative measures of animal health (fur condition, facial expression, food and water consumption) were observed daily during testing to ensure animal health (Sanz-Moreno et al., 2024; Burkholder et al., 2012). Animals were euthanized by overdose with isoflurane followed by cervical dislocation, and tissues were collected for future analyses. For this study, no animals were euthanized due to illness or injury for the duration of the experiments. Experiments were carried out in accordance with the National Institutes of Health Guide for the Care and Use of Laboratory Animals and received prior approval from the Indiana University Indianapolis School of Science Institutional Animal Care and Use Committee (IACUC; SC298R and SC338R).

### Locomotor activity

On P45, mice were placed in activity chambers with floor dimensions of 40.64×40.64 cm and plexiglass walls that were 30.48 cm high (Versamax, AccuScan Instruments, Inc., Columbus, OH, USA). Each chamber was equipped with an array with 16 equally spaced (2.5 cm) infrared beams across the length and width of the cage positioned (∼2 cm) above the chamber floor to detect horizontal movement. The chambers were fully enclosed in sound-attenuating cubicles with ventilation fans, limiting outside light and sound interference. Activity was monitored with a single 30-min session in the dark during the dark phase of the light:dark cycle. Measures recorded included total distance traveled (cm), time (s) the subject spent in the center of the chamber (defined as more than 8 cm from the walls, center area=36.7% of total area), absolute and percentage time in the center, absolute and percentage time in the margin (less than 8 cm from the walls), number of movements, time spent moving (s) and total rest time (s). The primary dependent variables were recorded in 1-min bins over the 30-min session. Prior to testing, chambers were thoroughly cleaned using 70% ethanol. Mice were transported to and from the testing room in their home cages, and a dark cloth was used to shield cages from light. Mice were allowed to habituate in the testing room for 10 min (with the covering cloth removed) to reduce the effects of novel environmental stress.

### Gait analysis

To assess gait on P47, mice were placed in an open-topped clear plexiglass corridor (57.15×8.25×15.24 cm). The corridor was positioned across two platforms (∼30.5 cm high) with a camera (Logitech C920 HD 1080p) positioned underneath the corridor for video imaging from a ventral view of the animal's movements (limbs and paws) during locomotion. The testing setup was housed in a dedicated testing room in the animal care facility with overhead red lighting and low white light (∼136 lumen/m^2^) provided by an LED bulb (8.5 W, 120 V, 60 Hz). White noise was supplied by a noisemaker (Yogasleep Dohm Classic, Model No. M1DSUSWH, 120 V, 60 Hz) to mask uncontrolled outside noise in the shared facility (e.g. from activity of individuals in the hallway outside the testing room). Gait was recorded for a single 5-min session during the subject's dark cycle. Prior to testing, and between each animal, the runway was cleaned thoroughly with 70% ethanol.

Videos were analyzed by identifying segments of uninterrupted forward movement on playback. Screenshots of individual hind paw placement during forward movement were captured and overlaid in Adobe Photoshop (2025 Computer software). Scores were generated masked to genotype but not sex. Approximately 20 overlays were produced per mouse, and ImageJ software (Schneider et al., 2012) was utilized to measure gait parameters (see Fig. S1).

### Balance beam task

The balance beam task was performed on P48-P49 during the dark phase in a testing room with overhead red lighting, using a previously published protocol (Luong et al., 2011) with modifications (Stringer et al., 2015; Roper et al., 2020). The apparatus consisted of 1 m painted wooden beams suspended between two metal poles using metal clamps ∼60 cm above floor level. A camera (Logitech C920 HD 1080p) was positioned at one end of the beam (the start location), and a dim white light was situated behind the camera (∼136 lumen/m^2^). A black acrylic box containing bedding from the home cage of the animals was placed at the opposite end of the beam to serve as a goal to motivate traversal of the beam. White noise was provided using a noisemaker (Yogasleep Dohm Classic, Model No: M1DSUSWH, 120 V, 60 Hz). Testing occurred during the dark cycle, and the beams and box were cleaned with 70% ethanol prior to testing and between animals.

Testing was conducted over 2 days for each cohort of mice, using three different beams with surface widths of 19, 12 and 9 mm. On the first day (designated as the training day to familiarize the mice with the task requirements), all mice of each cohort were first trained on the task using the 19 mm width beam, then all were subsequently trained with the 12 mm width beam. For the 19 mm beam training, a handful of home cage bedding was placed into the acrylic dark box, and mice were trained to traverse the length of the beam to the goal box. The mouse was first placed ∼5 cm from the goal box and then at increasing distances for subsequent trials, and encouraged to advance towards the dark box using light touch at the base of its tail. Once an animal had traversed the entire length of the beam twice without stopping, it was returned to its home cage for a rest period, the beam and box were cleaned with 70% ethanol, and new bedding was placed for the next mouse. After all mice of the cohort had completed training on the 19 mm beam, training ensued using the 12 mm width beam, following the same procedure.

On the second day (designated as the test day, on which mice were recorded using the Logitech camera), all mice of the cohort were first given three consecutive full-length trials traversing the 12 mm beam, followed by a rest period, then three consecutive full-length trials traversing the 9 mm beam. The videos were scored via playback at 0.25× speed by two trained independent scorers, masked to genotype, to quantify the number of hind paw slips, defined as either the left or right hind paw either slipping off or entirely missing the beam.

### Swim-escape adaptation

The swim-escape adaptation protocol utilized a 75-l glass fish tank (60.96×30.48×40.64 cm) filled with 25°C water made opaque with Crayola nontoxic white tempera paint (Blick Art Materials, Galesburg, IL, USA), fitted at one end with a white escape platform (9 cm diameter). Swim-escape training took place in a separate testing room from the MWM apparatus to ensure novelty of the MWM training environment at the start of visible platform training. Training was conducted under overhead red lighting with dim white light from a lamp source (∼136 lumen/m^2^). A noisemaker (Yogasleep Dohm Classic, Model No: M1DSUSWH, 120 V, 60 Hz) supplied ambient sound, and a camera (Logitech C920 HD 1080p) was used to record each trial. Training consisted of two visible platform trials followed by two hidden platform trials, for a total of four trials of swim-escape adaptation. For visible conditions, the vertical edge of the platform was wrapped with black electrical tape and situated ∼0.5 cm above the water level. For hidden trials, the tape was removed, and the platform was submerged ∼0.5 cm below water level. Latency to platform was recorded for each trial, and swimming capabilities of each animal were observed to assure adequate swimming to perform the subsequent MWM trials.

### Morris water maze task

The MWM task was conducted following a modified version of a previously published protocol (Wagner et al., 2014). As shown in Fig. S2, testing occurred over 12 consecutive days beginning at ∼50 days of age, and involved the following training sequence: (1) swim-escape adaptation (1 day, four trials); (2) visible platform acquisition of cued location learning (1 day, five trials); (3) submerged platform acquisition of place learning (7 days, four trials/day), with a single probe trial interposed at the start of the sixth place training day; (4) reversal training with the submerged platform (3 days, four trials/day).

The three phases of the MWM task (cued learning; place learning; reversal learning) were conducted using a 125 cm (diameter) pool positioned in the center of the testing room (3.38×2.82 m) and filled to within 25 cm of the top with water (∼25°C) made opaque with the addition of Crayola non-toxic white tempera paint (Blick Art Materials). Uniform room lighting was provided by dim indirect white light (∼117 lumen/m^2^) using four clamp lights fitted with 9.5 W LED bulbs, placed around the periphery and directed away from the tank. These lighting conditions optimized video tracking (Vorhees and Williams, 2006) and limited the potentially adverse effects of bright light on spatial performance (Huang et al., 2012). Various fixed visual cues, including wall posters, shelving and two black hanging curtains on either side of the pool that combined covered a ∼162° arc (45%) of the circumference, were placed around the outside of the tank. A white platform (9 cm diameter) was used for all MWM training. The computer-controlled tracking software (HVS Image, Hampton, UK) recorded *x*-*y* position every 100 ms and displayed the movement of the animal in the pool.

Cued training consisted of five successive trials with the visible platform location changed to a different quadrant for each trial using a random sequence. The vertical edge of the platform was wrapped in black electrical tape and placed ∼0.5 cm above the water level. Mice were tested in squads of three to four, resulting in an inter-trial interval of ∼3-4 min. For each trial, mice were placed in the pool at one of eight possible start points around the perimeter (avoiding the location closest to the platform), using the same random sequence of start points for each member of a squad but with different sequences across squads. Mice were given 60 s to locate and climb onto the platform, at which point they were allowed to remain on the platform for 10 s before being removed and placed in a heated incubator (30°C) to limit hypothermia. If an animal did not locate the platform within the 60 s period, the experimenter gently guided the mouse to the platform, allowed it to wait on the platform for 10 s, then removed it to the incubator. One squad that completed visible training had five mice (rather than the usual four). Following visible training of that squad, MWM testing was discontinued for one male euploid animal because the testing protocol for place and reversal training had a stringent requirement of no more than four mice per squad.

Starting the day after the visible training session (∼51 days of age), mice were given 7 consecutive days of acquisition training of place learning, with four trials per day. During these trials, the black electrical tape was removed from the platform, and the platform was submerged ∼0.5 cm below the water level. The platform location was assigned randomly to one quadrant for the duration of place training. Mice were released into the pool at one of seven possible start points (randomly assigned across trials, avoiding the start point closest to the platform). Start points were assigned using a random sequence for each member of a squad but with different sequences used across squads. Animals were given 60 s to locate the platform, then allowed to remain on the platform for 10 s before being removed to the incubator. On the first trial of the sixth day of place acquisition training, animals were given a 30 s probe trial, during which the platform was removed from the pool. The animal's search path was recorded and scored for spatial biases. Then, the four trials of the sixth day of place acquisition training were subsequently conducted. At the end of each day of training, the mice remained in their respective incubators for ∼10 min before being returned to the vivarium to minimize the effects of hypothermia.

On the day after the seventh day of acquisition training, animals began 3 consecutive days of reversal trials, with the submerged platform positioned in the opposite quadrant of the pool from its position in acquisition training. Four trials were conducted per day, and the start points were randomly assigned across trials. All other aspects of training were the same as for the place acquisition trials.

The HVS Image software superimposed four virtual counting discs in the center of each quadrant – 9 cm (diameter) for visible, acquisition and reversal training, and 27 cm (diameter) for the probe trials. These were positioned in the center of the four quadrants over the calibrated platform locations used for place training. Measures obtained during each training trial included latency to reach the platform (s), path length (cm), time spent within 11.36 cm of the wall (thigmotaxis), percentage time spent floating, heading angle (°) and swimming speed (cm/s; which excluded time spent floating). For the 30-s probe trials, measures included the path length, thigmotaxis, percentage time floating, heading angle, plus measures of time spent in, latency to enter and number of crossings through the four virtual counters.

### Statistical analyses

#### Data analysis approach

Data were analyzed using SPSS Version 31 (IBM Corporation). For each analysis, alpha was set to 0.05, and effect size metrics were reported as partial eta-squared values (η^2^_p_). The primary analyses of each dataset involved analyses of variance (either univariate ANOVAs or multivariate ANOVA) that included the two primary grouping factors of the study (genotype and sex) as fixed between-subjects factors. When appropriate, additional within-subjects factors were included as repeated measures in a RM-ANOVA. The assumption of equality of error variance across groups for a given set of data was only rarely violated (Levene's test). Departure from normality was evident (Q-Q plots and Shapiro–Wilk test) for measures of learning during MWM training (latency and path length) as training progressed over days and performance data became asymptote. Despite such departures, parametric statistics were used for all analyses, and the original scales of time and distance were retained, relying on the justification that ANOVAs are robust to such violations in terms of Type I error and transformation of data provide no additional benefits for control of Type I error (Blanca et al., 2017). For RM-ANOVAs, when the assumption of sphericity was violated in RM-ANOVAs, Greenhouse–Geisser corrections were used.

When a univariate ANOVA yielded a significant genotype×sex interaction, follow-up analyses of simple main effects of genotype were performed using linearly independent contrasts of genotype for each sex, to identify quantitatively the sex-specific effects of genotype contributing to the interaction (Keppel, 1982). Similarly, for data involving repeated measures, significant genotype×sex interactions were generally followed up with separate RM-ANOVAs on each sex. For some instances in which the main effect of genotype was significant but not the genotype×sex interaction (indicating that genotype effects were not significantly different between the sexes), we nevertheless conducted follow-up analyses within each sex using simple effects contrasts of genotype for each sex or (for repeated measures tests) separate RM-ANOVAS for each sex. The justification for these sex-specific analyses of genotype in the absence of a significant genotype×sex interaction was to characterize the extent to which the main effect of genotype was evident in the two sexes, and to make explicitly accessible the performance profiles of each sex in this novel mouse model.

#### Body weight

A three-way mixed ANOVA was conducted with age (P45, P51 and P62) as a within-subjects factor, and sex and genotype as between-subjects factors, with follow-up analyses of genotype×sex interactions using separate RM-ANOVAs for males and females followed by simple main effects contrasts. When within-group comparisons of body weights across the three ages were made, Bonferroni corrections for multiple comparisons were used.

#### Locomotor activity

Distance traveled (cm) and time in the center of the arena (s) were analyzed using a mixed-design RM-ANOVA with genotype and sex as grouping factors and time bin as a within-subjects factor. Significant and genotype×sex×time bin interactions were followed up using separate RM-ANOVAs for each sex with simple main effects analyses for genotype at each time bin. The genotype×time bin interactions were followed up with simple main effects contrasts of genotype (sexes combined) at each time bin.

#### Gait

The measures of the three gait parameters (stance width, stride length, step angle) were analyzed with a multivariate analysis of variance (MANOVA) with genotype and sex as between-subject factors. Significant multivariate effects were followed by univariate ANOVAs on each parameter, with follow-up simple main effects contrasts of genotype for each sex. In addition, stride length was analyzed with an ANCOVA with body length as a covariate, to control for potential effects of body length on that gait parameter.

#### Balance beam

The number of hind paw slips on the test day were analyzed using a mixed-design RM-ANOVA with beam width (12 mm and 9 mm) as a within-subject factor and genotype and sex as between-subject factors. Significant interactions were followed up with separate simple main effects contrasts.

#### Swim-escape adaptation

Latency to reach the platform was analyzed using mixed-design RM-ANOVA with training phase (visible versus submerged platform) and trial as within-subjects factor and genotype and sex as between-subject factors. Follow-up analyses were performed separately for each phase.

#### Morris water maze

For all MWM analyses, primary measures for each training phase were first analyzed with mixed-design RM-ANOVA with genotype and sex as between-subject factors, and trial (visible), day (place), quadrant (probe), or day and trial (reversal) as within-subject factors. The only training phase that yielded a significant genotype×sex interaction was reversal; follow-up analyses to identify sex-specific effects during reversal training were performed as described in the ‘Data analysis approach’ section. In the place acquisition training, each dependent variable was averaged across the four trials of each day of training, and those averages were then analyzed with day as the repeated measure in the RM-ANOVA. The probe trial data of time spent in the virtual counter in each of the four quadrants and the number of passes through each counter were analyzed using a mixed-design ANOVA with genotype and sex as between-group factors and quadrant as a within-subjects measure. To confirm the presence of significant probe trial search biases for each group, follow-up post hoc comparisons between the target and three non-target locations were conducted within each of the four groups using within-subjects comparisons with Bonferroni corrections. For the 3 days of reversal training, the initial analyses of the primary dependent variables used an RM-ANOVA with genotype and sex as between-subjects factors and day and trial as repeated measures. The presence of a significant genotype×sex interaction was followed up with separate RM-ANOVAs on males and females. When those sex-specific RM-ANOVAs yielded a genotype×(day×trial) interaction, simple main effects contrasts of genotype at each day/trial combination were used to identify the specific trials that contributed to sex-dependent differences in reversal learning between TcMAC21 and euploid groups responsible for the significant genotype×sex interaction.

## Supplementary Material

10.1242/dmm.053030_sup1 Supplementary information
