# Supplementary material for: Cognitive flexibility deficits differ between the sexes in a transchromosomic model of Down syndrome
Source: Dis Model Mech. 2026 Oct 28;19(10):dmm053030. doi: 10.1242/dmm.053030 (PMC13750394; doi:10.1242/dmm.053030)
Supplement: Supplementary file 1 [file dmm-19-053030-s1.pdf]

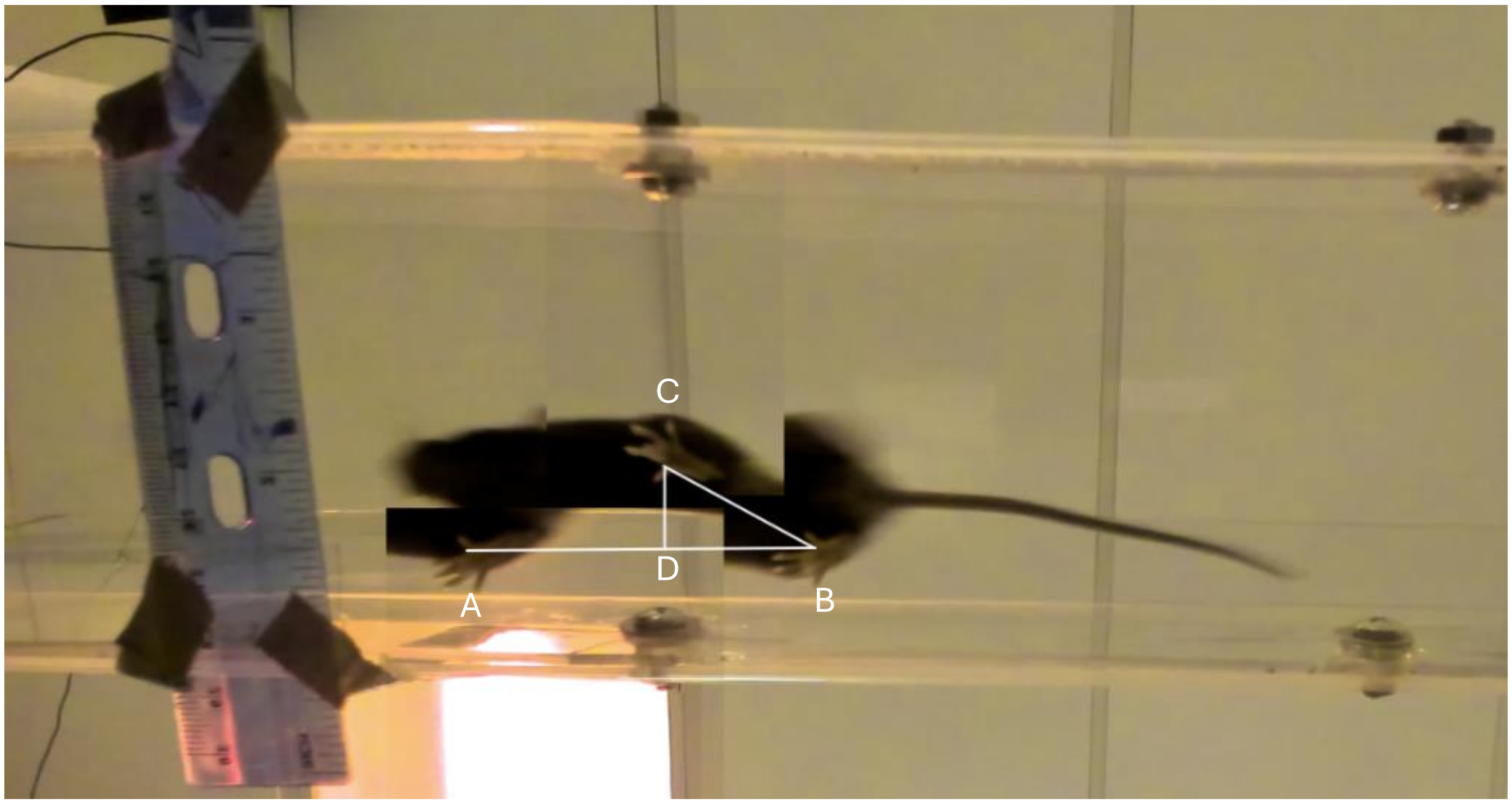

Fig. S1. Representative image of animal gait scoring using ImageJ. Parameters measured included stride length (A-B), stance width (C-D), step angle (A-B-C), and body length (tip of nose to base of tail).

Disease Models & Mechanisms • Supplementary information

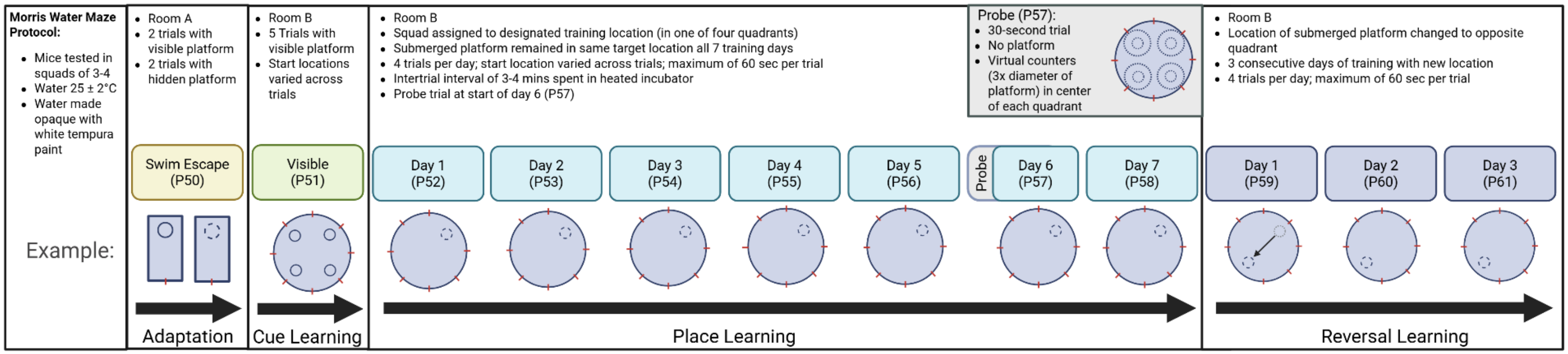

Fig. S2. Schematic of sequence and procedure for Morris water maze training protocol.

Disease Models & Mechanisms • Supplementary information

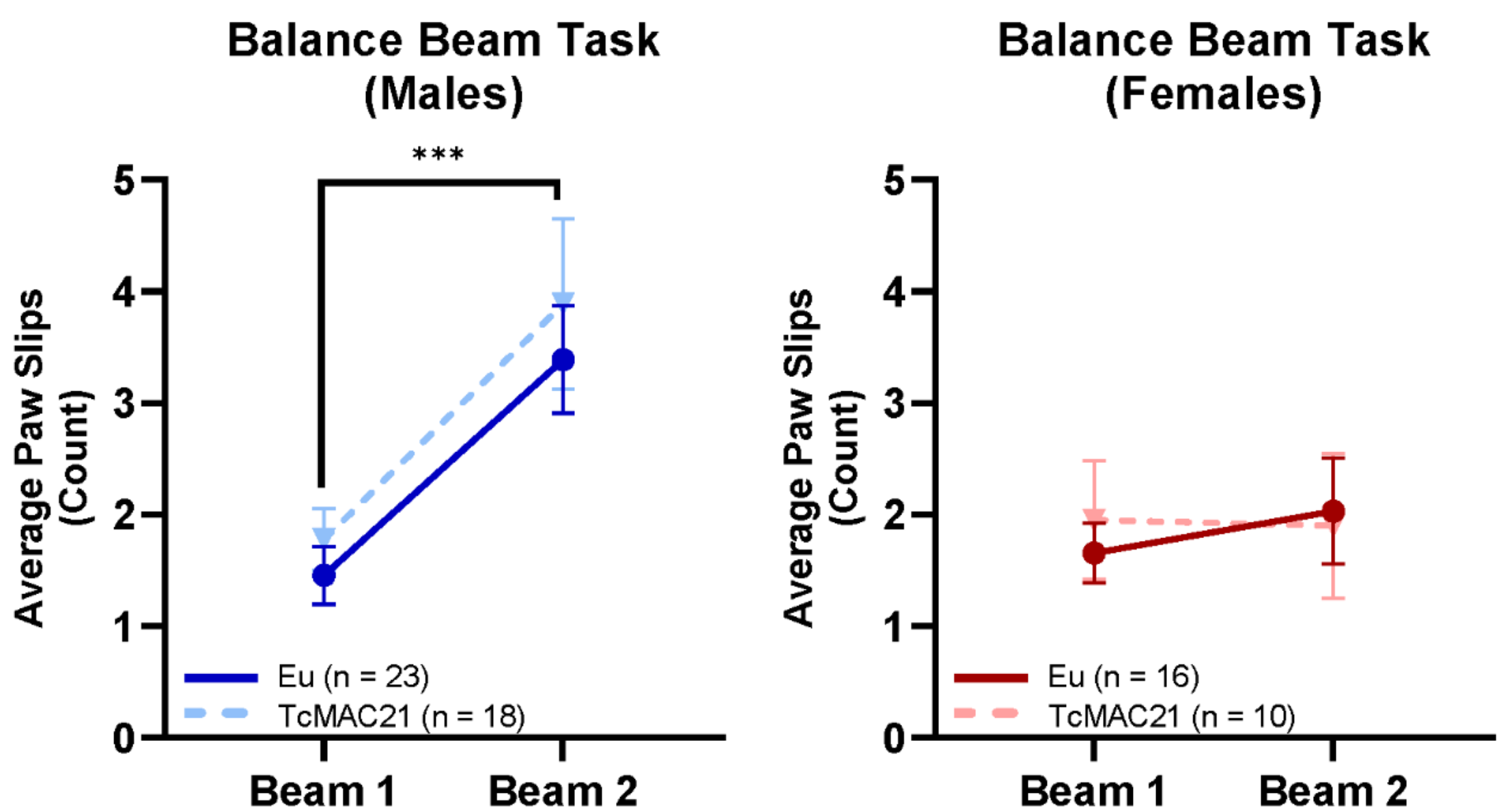

Fig. S3. Number of paw slips during the balance beam task (Mean ± SEM)**.** Three consecutive trials were conducted per beam. Sex differences in the effect of beam width were evident (significant sex × beam width interaction, $p < 0.001$; $\eta^2 p = 0.185$). Males showed a significant increase in paw slips due to the switch from beam 1 to the narrower beam 2; females showed comparable performance on the two beams. There was no significant main or interactive effect of genotype. ***p<0.001 simple main effects comparison between beam width within sex.

Disease Models & Mechanisms • Supplementary information

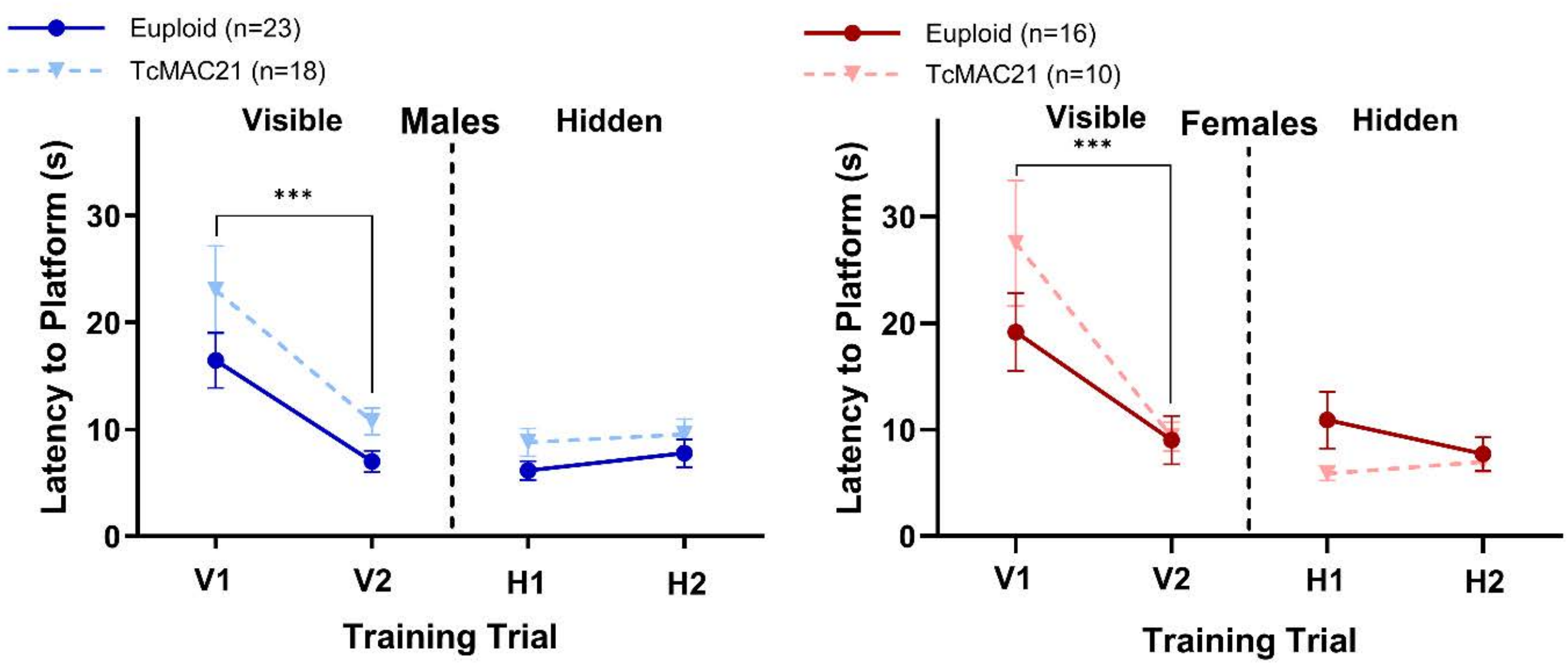

Fig. S4. Mean and SEM of latency to reach the platform in Swim Escape Adaptation trials. Significant differences were present only during the visible platform training (conducted first), yielding significant effects of phase ($p < 0.001$), trial ($p < 0.001$) and a phase × trial interaction ($p < 0.001$) on the RM-ANOVA. Follow-up RM-ANOVAs were performed separately for the two phases. These confirmed significant latency reductions from trial 1 to trial 2 during visible training (main effect of trial, $p < 0.001$), evident in both sexes of both genotypes, although TcMAC21 mice displayed longer latencies than euploid mice during visible training (main effect of genotype, $p = 0.037$). There were no significant main or interactive effects of sex. For hidden platform training, there were no significant main or interactive effects of any factor, consistent with all groups being near asymptote for escape performance on both trials.

Disease Models & Mechanisms • Supplementary information

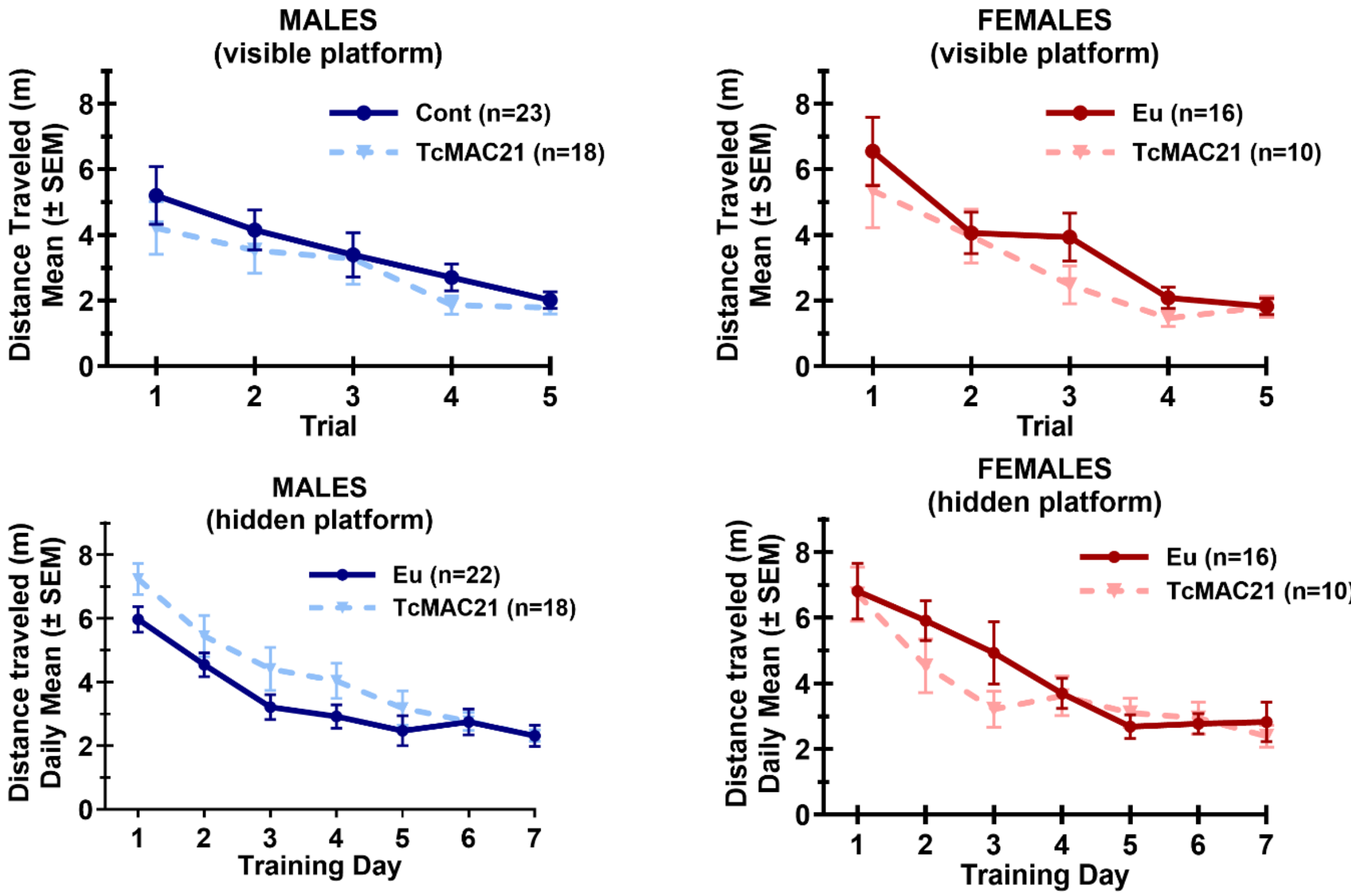

Fig. S5. Mean and SEM of distance traveled to reach platform for each of 5 trials of visible platform training (top panels) and for daily means of each of seven days of hidden (submerged) platform training. There were no significant main or interactive effects of genotype or sex on path length for either type of training. All groups showed significant reductions in path lengths over training both for cued ($p < 0.001$; $\eta^2_p = 0.210$) and place training ($p < 0.001$; $\eta^2_p = 0.450$). Error bars represent SEM.

Disease Models & Mechanisms • Supplementary information

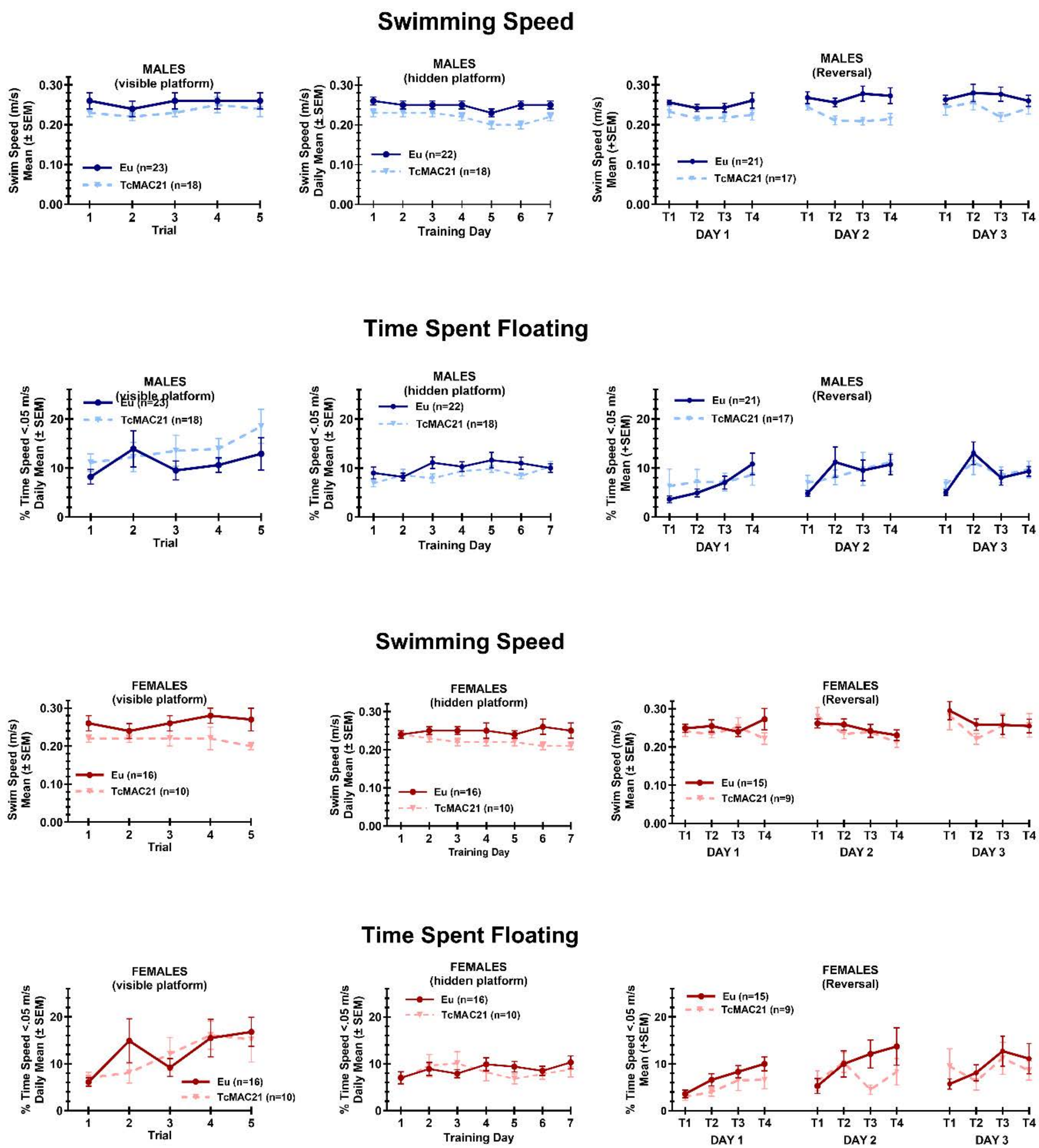

Fig. S6. Swimming speeds and percent time spent floating across visible, hidden, and reversal trials (Mean +/- SEM). Average swimming speeds were above 20 cm/s for both sexes across the three training conditions. TcMAC21 mice had modest but significant reductions in swim speed compared to euploid littermates on each training phase [main effects of genotype of $p = 0.025$ (visible), $p = 0.002$ (hidden), and $p = 0.037$ (reversal)], with overall average reductions of 11.3% (visible), 11.6% (hidden), and 10.4% (reversal). There were no significant main or interactive effects of sex for swimming speed for any training phase. For time spent floating, there were no main or interactive effects of genotype or sex during any training phase.

Disease Models & Mechanisms • Supplementary information

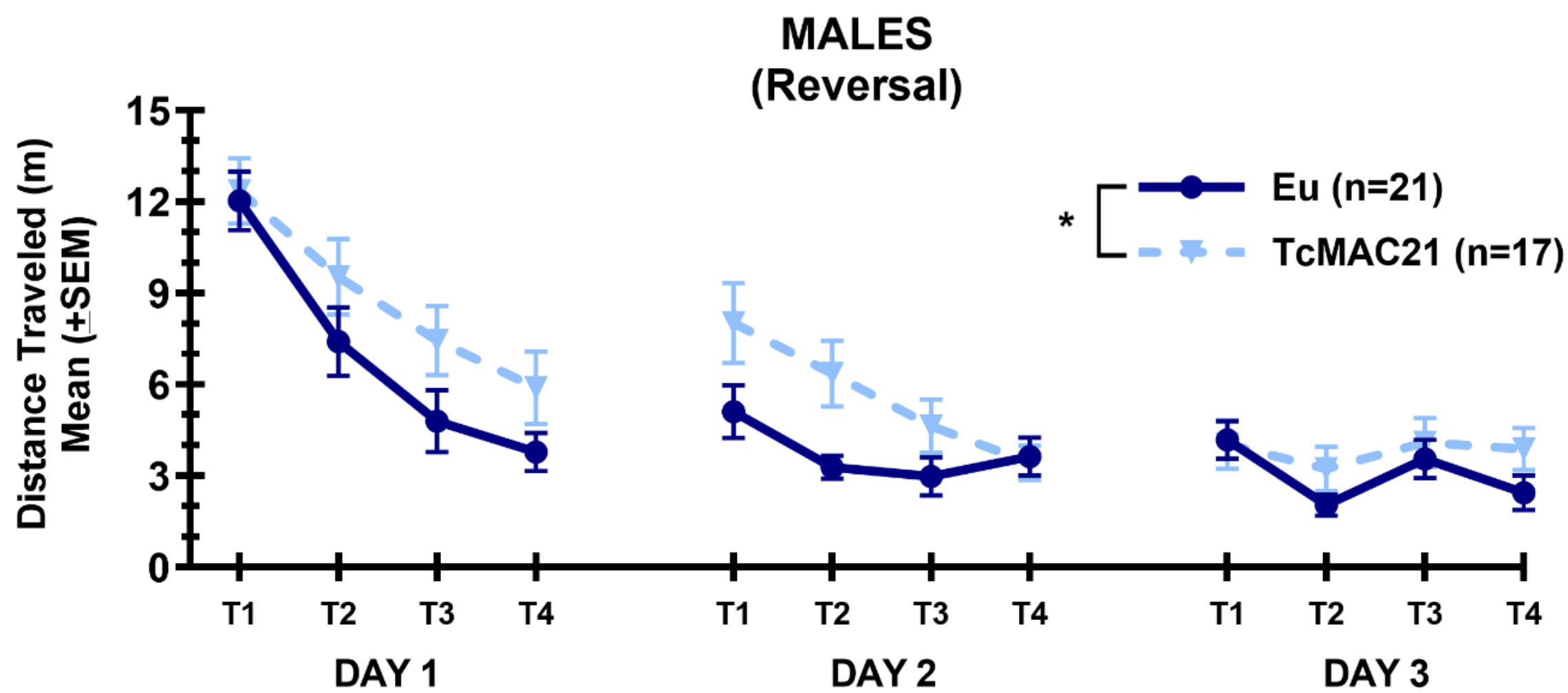

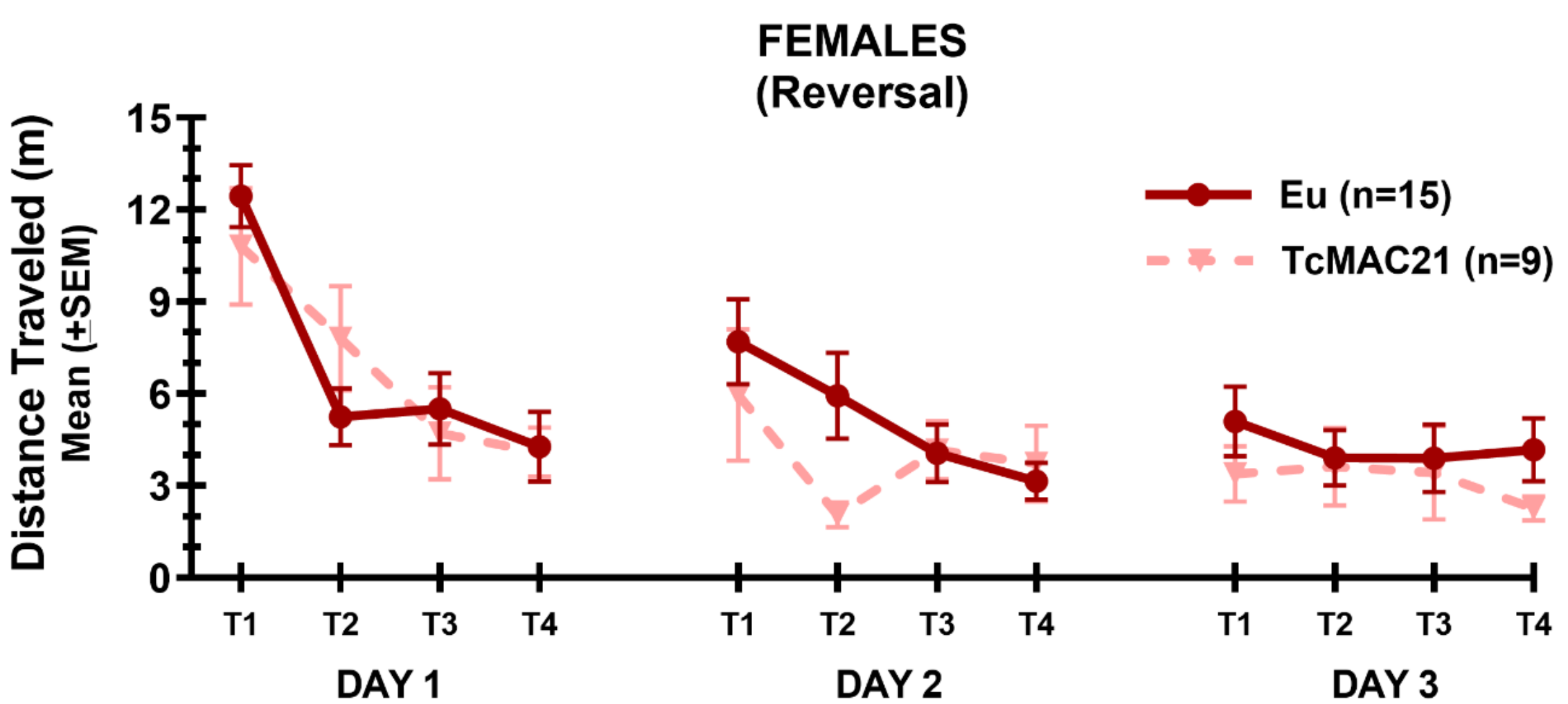

Fig. S7. Path lengths to reach the platform across three days of reversal training. Significant sex-specific impairments were evident during in reversal learning (genotype × sex interaction, $p = 0.032$; $\eta^2_p = 0.077$), with deficits in male TcMAC21 mice but not in female TcMAC21 mice. Follow-up RM-ANOVAs within each sex confirmed a main effect of genotype (TcMAC21 > euploid) in males (*p = 0.012), but no significant differences in females ($p = 0.373$).

Disease Models & Mechanisms • Supplementary information

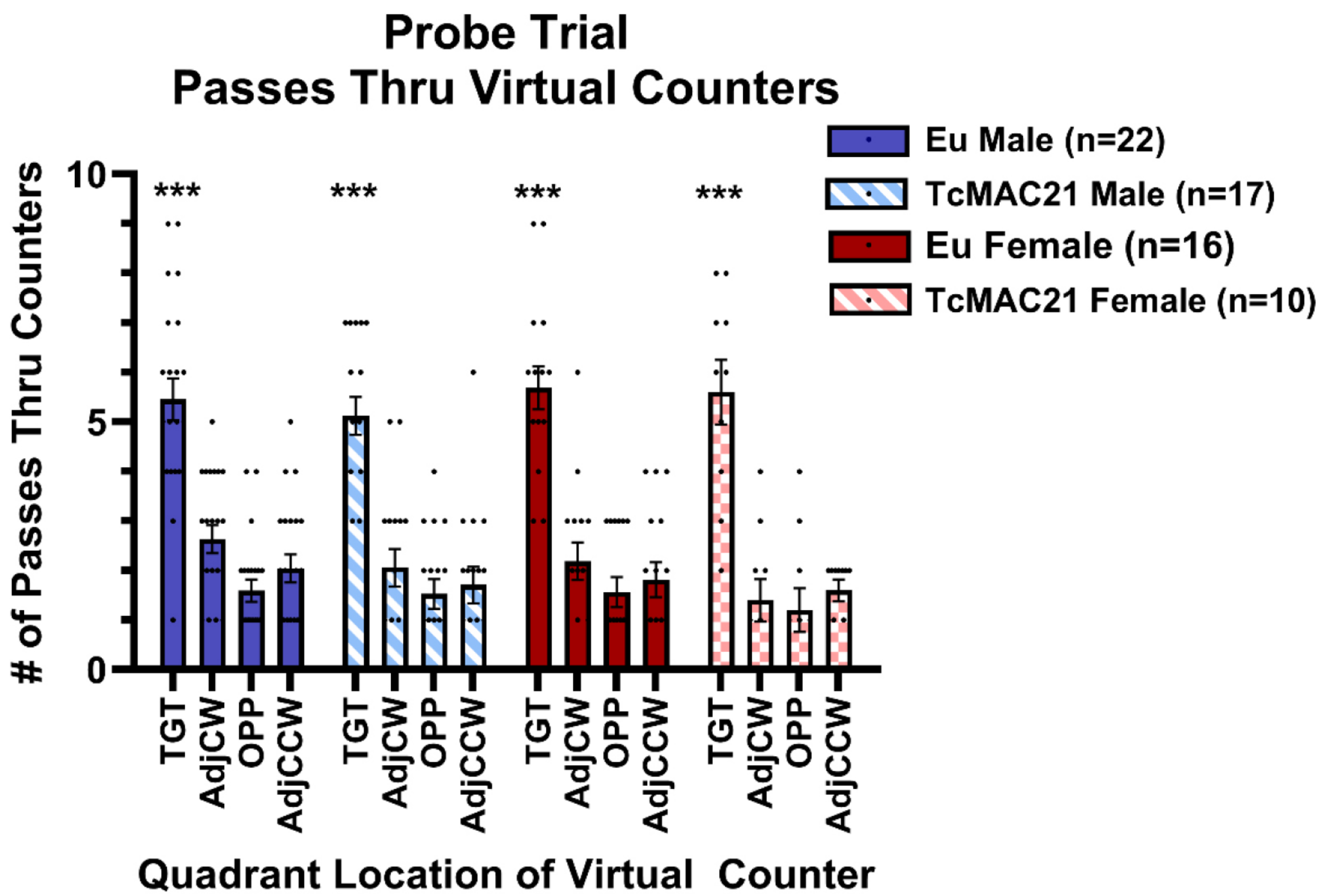

Fig. S8. Percent time passing through each of the four virtual counters during the probe trial. All groups spent significantly more passing through the counter in the target quadrant (TGT) than in the counters in the three non-target quadrants (AdjCW: adjacent clockwise; OPP: opposite; AdjCCW: adjacent counterclockwise). There were no significant main or interactive effects of genotype or sex.

Disease Models & Mechanisms • Supplementary information

Table S1. Additional measures from MWM testing

MWM Visible Training: Absolute Heading Angle (°), Mean +/- SEM							
**Genotype**	**Sex**	**N**	**Trial 1**	**Trial 2**	**Trial 3**	**Trial 4**	**Trial 5**
Euploid	F	16	66.2 +/- 7.4	57.1 +/- 10.1	52.8 +/- 11.1	41.7 +/- 7.4	40.0 +/- 6.2
TcMAC21	F	10	57.2 +/- 11.9	35.9 +/- 13.0	36.4 +/- 7.4	60.5 +/- 17.9	50.6 +/- 9.6
Euploid	M	23	57.4 +/- 7.3	47.8 +/- 6.4	45.5 +/- 4.1	52.0 +/- 6.5	31.8 +/- 5.8
TcMAC21	M	18	56.1 +/- 13.4	39.9 +/- 6.8	43.5 +/- 7.6	53.6 +/- 7.7	51.2 +/- 9.3

MWM Hidden Training: Thigmotaxis (% Time near wall), Daily Mean +/- SEM									
**Genotype**	**Sex**	**N**	**Day 1**	**Day 2**	**Day3**	**Day 4**	**Day 5**	**Day 6**	**Day 7**
Euploid	F	16	20.7 +/- 2.7	17.1 +/- 2.7	13.1 +/- 2.2	11.3 +/- 1.6	10.7 +/- 1.8	14.9 +/- 2.1	13.2 +/- 2.4
TcMAC21	F	10	22.6 +/- 2.5	14.9 +/- 1.9	10.8 +/- 2.6	15.6 +/- 2.5	17.1 +/- 3.3	18.3 +/- 3.1	16.5 +/- 3.0
Euploid	M	22	18.9 +/- 1.8	13.8 +/- 2.1	11.4 +/- 1.8	12.1 +/- 2.4	12.8 +/- 1.5	14.0 +/- 2.3	11.3 +/- 1.3
TcMAC21	M	18	24.2 +/- 2.4	15.2 +/- 1.7	15.8 +/- 2.1	14.1 +/- 1.5	12.0 +/- 1.9	14.0 +/- 1.8	15.3 +/- 2.2

MWM Hidden Training: Absolute Heading Angle (°), Daily Mean +/- SEM									
**Genotype**	**Sex**	**N**	**Day 1**	**Day 2**	**Day3**	**Day 4**	**Day 5**	**Day 6**	**Day 7**
Euploid	F	16	52.2 +/- 4.1	56.3 +/- 5.1	40.1 +/- 4.3	36.6 +/- 5.6	39.3 +/- 5.5	32.6 +/- 4.3	28.7 +/- 3.4
TcMAC21	F	10	48.5 +/- 6.8	42.7 +/- 2.4	45.3 +/- 7.3	41.2 +/- 5.1	34.9 +/- 6.3	35.7 +/- 5.0	33.1 +/- 5.3
Euploid	M	22	41.1 +/- 3.2	43.1 +/- 5.2	30.4 +/- 3.5	38.4 +/- 6.2	34.9 +/- 3.9	39.4 +/- 4.6	34.1 +/- 3.6
TcMAC21	M	18	46.4 +/- 3.8	44.3 +/- 3.4	41.4 +/- 3.6	35.0 +/- 3.4	30.8 +/- 3.9	34.5 +/- 3.5	34.1 +/- 2.7

Disease Models & Mechanisms • Supplementary information
